# Prioritizing policy issues for knowledge translation: a critical interpretive synthesis

**DOI:** 10.1186/s41256-025-00440-y

**Published:** 2025-08-20

**Authors:** Racha Fadlallah, Fadi El-Jardali, Tanja Kuchenmüller, Kaelan Moat, Marge Reinap, Mehrnaz Kheirandish, Lama Bou Karroum, Najla Daher, Nour Kalach, Lama Hishi, Gladys Honein-AbouHaidar

**Affiliations:** 1 Department of Health Management and Policy, American University of Beirut, Beirut, Lebanon; 2 Center for Systematic Reviews on Health Policy and Systems Research (SPARK), American University of Beirut, Beirut, Lebanon; 3 Knowledge to Policy (K2P) Center, American University of Beirut, Beirut, Lebanon; 4 Department of Health Research Methods, Evidence, and Impact (HE&I), McMaster University, Hamilton, Canada; 5 Research for Health, Science Division, World Health Organization, Geneva, Switzerland; 6 McMaster Health Forum/WHO Collaborating Centre for Evidence-Informed Policy, McMaster University, Hamilton, ON Canada; 7 Department of Health Evidence and Impact, McMaster University, Hamilton, ON Canada; 8 Division of Country Health Policies and Systems, WHO Regional Office for Europe, Copenhagen, Denmark; 9 Department of Science, Information and Dissemination, WHO Regional Office for the Eastern Mediterranean, Cairo, Egypt; 10 Hariri School of Nursing, American University of Beirut, Beirut, Lebanon

**Keywords:** Critical interpretive synthesis, Priority setting, Knowledge translation, Evidence-informed policymaking, Prioritization of issues, Conceptual framework

## Abstract

**Background:**

While calls for promoting evidence-informed policymaking (EIP) have become stronger in recent years, there is a paucity of methods to prioritize issues for knowledge translation (KT) and EIP. As requested by WHO and as part of efforts to address this gap, we conducted a critical interpretive synthesis (CIS) to develop a conceptual framework that outlines the features of priority-setting processes and contextual factors influencing the prioritization of issues for KT efforts.

**Methods:**

We systematically reviewed the literature and used an interpretive analytic approach—the CIS—to synthesize the results and develop the conceptual framework. We used a "compass" question to create a detailed search strategy and conducted electronic searches to identify papers based on their potential relevance to priority-setting for KT efforts and EIP.

**Results:**

We identified 161 eligible papers. Our findings on key features of the priority-setting process unpacked three 3 levels of constructs: ‘pathways’ for identifying and prioritizing policy issues for knowledge translation efforts; ‘phases’ within each pathway; and ‘steps’ for each phase. There are three main pathways: (1) explicit and systemic priority-setting processes involving policymakers and stakeholders to determine priority topics (collaborative); (2) a policymaker or stakeholder brings an issue forward or asks for evidence on a particular topic (demand-driven); and (3) a need or policy gap is identified by a knowledge translation platform (supply-driven). Within each pathway, four phases emerged: “Preparatory”, “prioritization”, “knowledge translation” and “scale-up and sustainability”. Across these phases, the following steps were identified: establishing a core team, defining a scope, confirming a timeline, sensitizing stakeholders, generating potential issues, gathering contextual information, setting guiding principles, selecting prioritization criteria, applying the method for prioritization, documenting and communicating priorities, validating and revising priorities, selecting venue for decision-making, implementing priorities, monitoring and evaluation, promoting institutionalization, and engaging in peer learning and exchange of experience. We identified engaging stakeholders and strengthening capacity as cross-cutting elements. Our findings on contextual factors unpacked four categories: (1) institutions; (2) ideas; (3) interests; and (4) external factors.

**Conclusions:**

This CIS generated a multi-level conceptual framework for prioritizing issues for KT efforts and laid the foundation for a WHO tool that supports prioritization in practice. The study contributes meaningfully to both the literature and the operationalization of KT and EIP.

**Supplementary Information:**

The online version contains supplementary material available at 10.1186/s41256-025-00440-y.

## Background

In recent years, there has been growing movement to ensure that policymaking processes are informed by the best available evidence [1–3]. Despite increasing efforts to promote evidence-informed policymaking (EIP), the research to policy gap still persists [4, 5]. Knowledge translation (KT) is a term often used in health research, policy, and practice settings to describe the activities and processes needed to facilitate evidence‐informed decision‐making and enhance the use of research evidence [6]. The World Health Organization (WHO) defines KT as the “synthesis, exchange, and application of knowledge by relevant stakeholders to accelerate the benefit of global and local innovation in strengthening health systems and improving people’s health” [7]. A starting point for KT is the identification of a policy-relevant issue (problem, topic or concern) within a particular health system [8, 9].

Decision-makers and organizational knowledge brokers are faced with a growing number of competing policy issues under limited time and resource constraints. Prioritizing policy issues for KT is essential to ensure optimal allocation of limited resources towards issues that are relevant and likely to impact policy or practice [10–12]. A structured priority-setting process enables a more strategic planning, increasing the likelihood of evidence utilization in policymaking [12, 13].

There is a paucity of methods that governments and organizational knowledge brokers can use to prioritize policy issues for KT products and EIP. Existing systematic reviews examined approaches and frameworks to prioritize topics or questions for systematic reviews [14], practice guidelines [15] or primary health research [16]. We are not aware of any existing priority-setting framework or approach for KT products which take into consideration the process, the organizational attributes or features, and the contextual factors that affect the process. The development of KT products pose different conceptual and methodological challenges related to the varied types of evidence that are relevant, valued, and considered; the complexity of health systems’ internal and external relations that must be addressed; and the pre-eminence of contextual factors (e.g., long-standing health-system issues versus emergent issues, contestable versus salient issues, etc.) [17] that directly influence the design and adoptability of products [18].

Following the WHO's request to develop a framework and tool to support countries in creating impactful KT products that address policymakers’ needs, a knowledge synthesis of the literature on the key features of and approaches to prioritizing policy issues for KT efforts was conducted. The sub-questions explored were: What are the key features of a priority-setting process and the approaches used to prioritize issues for KT efforts? What contextual factors influence this prioritization?

## Methods

### Design

We conducted a critical interpretive synthesis (CIS), which is a particular form of systematic review that combines qualitative research inquiry with systematic review methodology to analyze a broad range of relevant sources and use analytical outputs to develop a conceptual framework [19, 20]. CIS is the most appropriate knowledge synthesis approach for studying emerging, ill-defined phenomena [21, 22]. We chose CIS as it suited the nascent body of literature on prioritizing policy issues for evidence-informed decision-making, aiming to generate key constructs and domains for the KT priority-setting process. As per CIS methodological standards, our review questions served as compasses rather than anchors, allowing the concepts to be derived from synthesis of the literature and iteratively modified throughout the review [19, 20]. An internal protocol for this synthesis work is available upon request.

### Literature search

We searched the following electronic databases in February 2022: Medline, EMBASE, CINAHL, ProQuest, and Global Health Library. We also searched Health Systems Evidence, Social Systems Evidence, and Google Scholar. We used both index terms and free text words related to two concepts: ‘priority setting’ and ‘policy or knowledge translation’. The team manually developed the search strategy (no published search filters were used), which was validated by an information specialist (see Additional file 1).

Two rounds of additional purposive sampling were conducted in June 2022 and May 2023 to help refine the conceptual framework. The additional articles were purposively sampled by content experts and by searching for specific concepts that emerged from the first round of analysis, and that were important theoretically and needed to fill conceptual gaps. We stopped sampling at the saturation point where looking at new literature no longer contributed additional concepts/constructs or additional descriptions of the identified concepts. Additionally, we searched the websites of selected KT platforms (McMaster Health Forum, Knowledge to Policy (K2P) Center, and the Center for Rapid Evidence Synthesis (ACRES)) and international organizations (e.g. the WHO Evidence-informed Policy Network (EVIPNet)) for relevant documents. We also conducted an analytical review of KT products (evidence briefs for policy and rapid response products) sampled from these sources in May 2023. We selected ten KT products per institution, published within the last ten years, ensuring representativeness across sectors (e.g. health, social, etc.), systems-level focus (governance, financing, delivery), and population (e.g. elderly, etc.). We analyzed the problem section of each KT product to gain additional insights into how issues were prioritized, the features of the prioritization process and the contextual factors influencing the process. We sought a clear framing of these factors by drawing on concepts related to political context—including institutional, interest-group, and idea-related factors well established in political science literature—while remaining open to other relevant emerging factors [17].

### Eligibility criteria

We included studies on frameworks, models, theories, or approaches for prioritizing issues for KT and EIP, as well as those evaluating prioritization processes, or reporting on contextual factors influencing prioritization or KT product development. In anticipation of the potentially limited literature on prioritizing policy issues for KT efforts/products, we also included studies on broader policy prioritization and health systems research for their conceptual contributions. Both empirical and conceptual studies were eligible, with no restrictions on language, country, or sector. We prioritized inclusion for ‘highly relevant’ records first, before moving to others that were not as relevant (e.g., as a way to consider more conceptual contributions to the analysis). We excluded studies solely focused on prioritization of clinical research, clinical practice guidelines, healthcare interventions or KT in clinical settings (unless linked to national decision-making). We also excluded HTA, economic evaluations, and KT frameworks lacking an explicit description of the priority-setting component.

Quality appraisal was not conducted, as our goal was to develop a conceptual framework based on insights from relevant literature rather than specific quality criteria.

### Selection process

We conducted a calibration exercise using a selected sample of 199 articles to pilot and refine the eligibility criteria. Reviewer agreement was assessed using the kappa statistic.

We adopted a phased-approach to study selection. In phase 1, we applied exclusion criteria to titles and abstracts of retrieved articles to remove those that were obviously not relevant to the purpose of our study. In phase 2, we screened the full text of ‘potentially relevant’ studies, to determine which ones got into the analysis. A schema was constructed to select relevant papers from the pool of ‘potentially relevant’ studies through purposive sampling [17]. First, we used a purposeful sampling approach to explore the literature to determine the range of unique candidate concepts (or theoretical domains) associated with prioritizing policy issues for KT products (or policy issues in general). We stopped sampling at the saturation point where looking at new literature no longer contributed additional concepts. Second, we employed a theoretical sampling strategy to analyze the literature relevant to each of the identified concepts. Articles directly addressing the prioritization of issues for KT efforts or EIP were included, supplemented by purposively sampled articles from the broader literature (e.g., prioritization of policy issues in general) for their potential conceptual contribution to our review. The aim was to capture the thematic depth of the concept across the literature through inductive and iterative conceptual analysis rather than collect numerous citations of repetitive concepts and/or descriptions [23].

### Data abstraction and synthesis

As per CIS methodology, we gave latitude for a more narrative data retrieval approach [21, 23]. We extracted key elements (using Excel sheet) from all the papers, including author, year of publication, country, purpose, relevance, and a summary of its main conceptual contributions.

Data was synthesized interpretively using the constant comparative analysis approach to ensure that the emerging synthesized constructs are grounded in the data. For the framework development, we started by identifying common themes and concepts, with greater attention given to those emerging from multiple documents that help to clarify the process and key attributes/features of priority-setting for KT efforts [24].

We also explored political effects on priority-setting, using the interpretive concepts of institutions, interests, and ideas to understand real-world decision-making and resource allocation experiences [25]. The identified constructs were integrated to produce a synthesized conceptual framework.

## Results

**Fig. 1:**
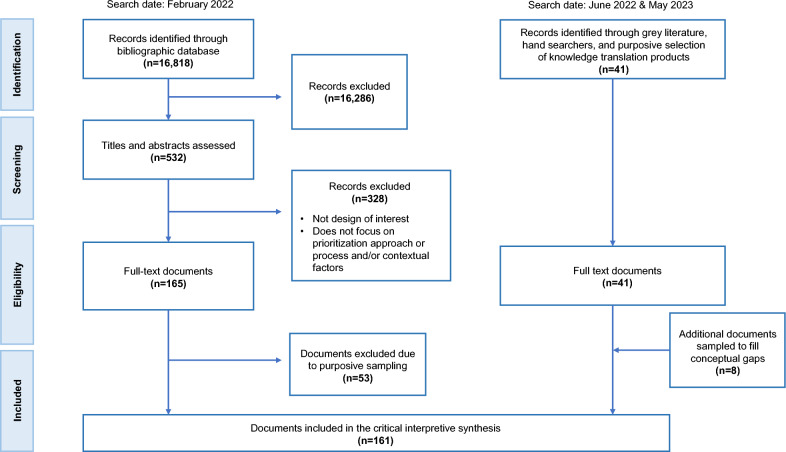
PRISMA flow chart for study selection process

Figure 1 presents the PRISMA flow diagram of the study selection process. Of 16,867 citations identified, 161 articles were included in the final synthesis (the full list of references is available upon request). The Kappa statistic was 0.62 on the samples for the first step, and 0.75 for the second indicating acceptable inter-reviewer agreement.

The characteristics of the 161 papers, published between 1994 and 2023, are summarized in Table 1. Key priority-setting features (e.g., principles, frameworks, tools) were addressed in 37 (23%) papers, contextual factors (e.g., political landscape, stakeholder interests) in 112 (70%), and both aspects in 12 (7%). Studies primarily came from high-income countries (n = 70, 43%) and lower-middle-income countries (n = 67, 42%). Methodologically, 79 were empirical, 41 were classified as ‘policy briefs,’ 33 were conceptual and eight were a mix of both empirical and conceptual. The majority of studies focused on health policy (n = 129, 80%).

**Table 1:** Characteristics of included studies

Characteristics		N	%
Peer-reviewed vs. Gray literature	Peer-reviewed	111	67
	Gray literature	50	33
Type of study	Empirical	79	49
	Policy Briefs	41	25
	Conceptual	33	21
	Both	8	5
Income Level*	High income	70	43
	Upper middle	43	27
	Lower middle	67	42
	Low income	21	13
	More than one**	10	6
	N/A	17	11
Discipline	Health policy (incl. health services and policy research and health economics)	129	80
	Social policy/public administration/political science	27	17
	International development	4	2
	Other	1	1
Arrangement ^†^	Delivery	14	11
	Governance	13	10
	Financing	11	8
	More than one	42	33
	Not specified	49	38
Sector (health and non-health)	Public health	39	24
	Primary Care	7	4
	Multisector	7	4
	Specialty care	5	3
	Home and community care	3	2
	Long-term care	2	1
	Rehabilitation care	1	0.6
	Other	3	2
	Not specified	94	58.4
Prioritization	Formal (without an underpinning logistical or theoretical framework)	39	24
	Formal (model/theory/framework)	9	6
	Informal	3	2
	Both	2	1
	N/A	108	67
Level of prioritization ^β^	National	14	26
	Regional	12	23
	International	10	19
	Sub-national (district, provincial or municipal)	10	19
	Global	7	13
Conceptual contribution	Contextual factors	112	70
	Process of prioritization	37	23
	Both	12	7

^*^The total count and percentage may not equal 161 and 100%, respectively, as some studies fall into multiple categories

^**^Studies included diverse geographical contexts, representing countries across different income levels

^†^Denotes studies solely focused on the health policy discipline

^β^Denotes studies solely focused on the prioritization element

### Conceptual framework: key constructs for priority-setting for knowledge translation efforts

**Fig. 2:**
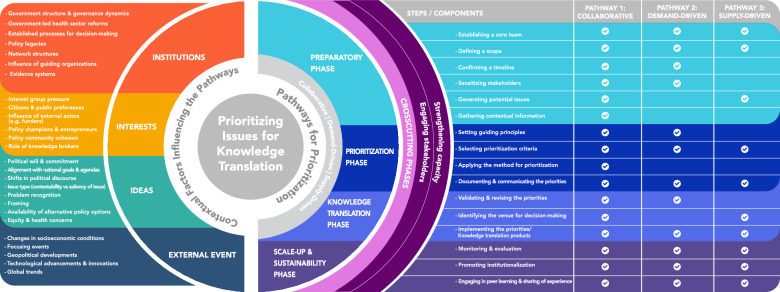
Conceptual framework for prioritizing policy issues for knowledge translation efforts

Our analysis identified key constructs encompassing both the features of priority-setting processes and the contextual factors influencing the prioritization of issues for KT and EIP. The resulting conceptual framework that emerged from our analysis outlines these constructs, highlighting how priority-setting for KT unfolds and the contextual factors that shape the process (Fig. 2).

The framework centers on three *pathways* for identifying and prioritizing policy issues for KT efforts: (1) explicit and systemic priority-setting processes involving policymakers and stakeholders to determine priority topics (collaborative); (2) a policymaker or stakeholder brings an issue forward or asks for evidence on a particular topic (demand-driven); and (3) a need or policy gap is identified by a KT platform (supply-driven). The framework also outlines four *phases* within each pathway (preparatory, prioritization, knowledge translation and scale-up and sustainability), and within each phase 18 unique *steps/components* (e.g., establishing a core team, defining scope, selecting prioritization criteria). Table 2 provides a summary of the how each of the identified phases and steps contributes to our understanding of the three prioritization pathways.

**Table 2:** Pathways, phases and steps influencing priority-setting process for knowledge translation efforts

Phases	Steps	Pathways		
		Pathway 1: collaborative	Pathway 2: demand-driven	Pathway 3: supply-driven
Preparatory phase	Establishing a core team	**✓**	**✓**	**✓**
	Defining a scope	**✓**	**✓**	**✓**
	Confirming a timeline	**✓**	**✓**	
	Sensitizing stakeholders	**✓**	**✓**	
	Generating potential issues	**✓**		**✓**
	Gathering contextual information	**✓**		
Prioritization phase	Setting guiding principles	**✓**	**✓**	
	Selecting prioritization criteria	**✓**	**✓**	**✓**
	Applying the method for prioritization	**✓**		
	Documenting and communicating priorities	**✓**	**✓**	**✓**
Knowledge translation phase	Validating and revising the priorities	**✓**	**✓**	
	Selecting the venue for decision-making	**✓**		**✓**
	Implementing the priorities/ KT products	**✓**	**✓**	**✓**
Scale up and sustainability phase	Monitoring and evaluation	**✓**	**✓**	**✓**
	Promoting institutionalization	**✓**	**✓**	**✓**
	Engaging in peer learning and exchange of experience	**✓**	**✓**	**✓**
Cross-cutting constructs	Engaging stakeholders	**✓**	**✓**	**✓**
	Strengthening capacity	**✓**	**✓**	**✓**

In the sections that follow we provide additional details about the core components of the conceptual framework outlined above (Fig. 2) and summarized in Table 2. We also briefly explain how four *contextual categories* identified in our analysis (institutions, interests, ideas and external factors) influence the prioritization of issues for KT by pushing issues higher (or lower) on a policy agenda, or by influencing one of the three pathways.

### Pathways, phases and steps influencing priority-setting process for knowledge translation efforts

As noted above, the theoretical framework developed through our analysis centers on three key pathways for identifying and prioritizing policy issues for KT efforts, and within them distinct phases and steps (Table 2).

#### Pathway 1: Collaborative

Pathway 1 was labelled ‘collaborative’ given it involves explicit and systemic priority-setting processes involving policymakers, other stakeholders and researchers/KT platforms to determine priority topics, and encompasses four key phases: preparatory, prioritization, KT, and scale-up and sustainability.

The preparatory phase for Pathway 1 includes six distinct steps. The first is establishing a core team with technical expertise and decision-making authority to oversee the process that can help initiate plans for leadership, logistics, budget and communication strategies early in the process [26]. The second is defining the scope and focus of the priority-setting exercise (e.g. by disease area, programmatic or systemic priorities, geographical reach or periodicity), often informed by a comprehensive situation analysis exercise, environmental scan and/or assessment of available resources and health systems environment [27, 28]. The third is establishing a timeline for which the priorities are expected to be valid or relevant, as priorities may evolve due to changes to the health system or socio-political contexts, emerging evidence, or technological advancements [29]. The fourth step in the preparatory phase of Pathway 1 is stakeholder sensitization, which could include efforts such as workshops early in the process (which may empower stakeholders and enhance their active participation, galvanize support for the initiative and strengthen partnerships that positively contribute to the prioritization process) [30–34]. The fifth step is generating potential issues for prioritization (e.g., through document reviews, stakeholder consultation or situation analysis), which can vary in terms of level of detail required, depending on the focus of the prioritization exercise (e.g., themes vs. topics. vs. targeted policy questions), and can be driven and supported by strategically positioned policy entrepreneurs [14, 28, 29, 31, 35–43]. Finally, the sixth step of the preparatory phase for Pathway 1 is preparing a pre-circulated summary of available data and contextual information, which ensures participants are well-prepared for deliberations on potential issues for prioritization and have a level playing field/common understanding before deliberating about the issue(s) at hand [11, 30, 31, 39, 44, 45].

The prioritization phase for Pathway 1 includes four key steps. The first step of this phase is setting guiding principles, which is important for the process to be perceived by all stakeholders as fair and legitimate, and can include things like drawing on research evidence, transparency and inclusiveness [30, 46]. The second step in the prioritization phase for Pathway 1 is selecting prioritization criteria to communicate rationale behind priorities, and to promote objectivity, transparency and validity of the process (available upon request is a list of 36 unique prioritization criteria across seven domains identified during the analysis). The third step in the prioritization phase of Pathway 1 is choosing and applying the prioritization method, which requires a systematic and transparent approach—ideally one that aims for fair representation of those involved in or affected by the issue under consideration, engages a skilled and neutral facilitator, and applies explicit prioritization criteria within consensus-based, metrics-based, or hybrid approaches [11, 28, 35, 46]. The fourth and final step in the prioritization phase of Pathway 1 is documenting and communicating priorities to enhance credibility and acceptability as well as improve awareness, uptake and implementation of the priorities in forms that are suitable for use in KT products [11, 37, 42, 44].

The knowledge translation phase of Pathway 1 includes three steps. The first step is validating and revising priorities, which ensures they remain relevant given the context and intended audience and viewed as credible [29, 30, 37, 47–49]. The second step is identifying venue for decision-making (e.g., Ministry of Health), which may be involved in one or more level (e.g., national or sub-national), and will influence how a KT product is tailored to reflect the appropriate format and language required by the target audience and context [47]. The third and final step of the knowledge translation phase of Pathway 1 is implementing the priorities/KT products—with the most common type being evidence briefs for policy prepared to inform policy dialogues on priority issues [14, 31, 32, 47, 50, 51]. To be done successfully, this requires stakeholder support, mobilizing funding, assigning responsibilities, and collaborating with researchers and KT platforms to develop proposals and products [37, 46, 48].

The final phase in Pathway 1—scale-up and sustainability—while relatively underdeveloped in the literature identified in this study, includes three steps. The first step focuses on integrating monitoring and evaluation plans (inclusive of both process and outcomes measures) into the priority-setting cycle to support continuous improvement, potential for scaling up, commitment, and capacity building for future processes [11, 14, 29, 46]. The second step is promoting institutionalization of the process (e.g., through legal frameworks, governance structures and capacity-building efforts) to ensure it is routinized and sustained, rather than a ‘one-off’ exercise [43, 52–56]. The third step in the scale-up and sustainability phase of Pathway 1 is engaging in peer learning and exchange of experience (e.g., conferences) to foster continuous improvement and further institutionalization of the process [13].

#### Pathway 2: Demanddriven

Pathway 2 was called demand-driven given it involves a policymaker or stakeholder bringing an issue forward or asking for evidence on a particular topic. The majority of studies identified in this category focused specifically on establishing rapid-response services, and provided insights about how key aspects of those programs would also be relevant to demand-driven priority-setting processes. Our analysis indicated that demand-driven prioritization processes are often triggered when the demand for evidence exceeds perceived supply, or when a decision-maker needs support prioritizing multiple issues. To facilitate this process, a central structure to receive and prioritize requests is essential as is maintaining regular contact with requesters. There were many overlaps in the four phases and steps within them identified for Pathway 1 (see Table 2), so the focus of the summary below is on the unique aspects for Pathway 2.

The preparatory phase for Pathway 2 includes four steps. The first step is establishing a core team, which occasionally takes the form of a steering committee, and which can support building trust, ensuring quality and credibility and promoting the uptake of the outputs of demand-driven prioritization processes. The second step of this phase for Pathway 2 is defining scope and focus, including specifying the field (e.g., health vs. social), questions to be answered (e.g., clarifying the problem vs. identifying solutions), and the anticipated volume of requests that can be responded to (which also includes managing number of requestors that can be engaged to balance capacity to respond with anticipated demand) [29, 46, 57–61]. The third step is establishing clear timelines for request completion, and the level of support provided within various timelines to manage expectations [11, 40, 45, 59, 60, 62]. The fourth step as part of the preparatory phase of Pathway 2 is sensitizing stakeholders, which includes helping them to recognize and value research as an input in policymaking, thus creating demand for it (e.g., through advocacy and reminders) [59].

The prioritization phase for Pathway 2 included three steps. The first step is setting guiding principles which requestors should adhere to (e.g., basing findings on evidence rather than personal views and identifying conflicts of interest) [58]. The second step as part of the prioritization phase for Pathway 2 is selecting prioritization criteria to guide the process (e.g., prioritizing infrequent over frequent requestors, addressing urgent topics first, utilizing an explicit ranking process to make decisions about which requests to prioritize), which should take into account key factors such as timelines, and also include maintaining an inventory of pending, active and completed requests [11, 58]. The third step in the prioritization phase of Pathway 2 is documenting and communicating the process, with details such as request types, applied criteria and outputs identified as the most important factors to document for accountability [59, 60], and with the importance of a communication strategy (subject to confidentiality provisions) also emerging as necessary to inform policymakers and stakeholders about high-priority issues, while enabling them to contribute to problem clarification, framing options and addressing implementation considerations [11].

The knowledge translation phase of Pathway 2 includes two steps. The first step is validating and revising priorities, which includes reviewing topic ranking at regular intervals (facilitated by team leads and with a focus on clarifying the rationale for any changes to priorities) while balancing timelines and comprehensiveness of outputs given available resources. The second step is implementing the priorities/ KT products, which typically involves dissemination efforts (e.g., delivering products to end users and/or making it publicly available).

The final phase of Pathway 2—scale up and sustainability—included three steps. The first step was monitoring and evaluation, and was framed as a key aspect of tracking progress towards addressing demand (including when high-priority issues were not responded to, despite demand) [46, 57, 62], and systematically examining how and why stakeholders use the outputs and the impacts on policymaking processes [11]. The second and third steps of the scale up and sustainability phase of Pathway 2 (institutionalization and peer learning) align with those outlined in this phase for Pathway 1.

#### Pathway 3: Supply-driven

Pathway 3 focuses on prioritization of policy issues for KT is driven by a KT platform, and was the least documented pathway for prioritizing policy issues for KT efforts. This likely reflects its largely implicit and unstructured nature. Despite this, our analysis suggested that Pathway 3 includes the same four phases as Pathwayas 1 and 2: preparatory, prioritization, KT, and scale-up and sustainability, and furthermore, that it is typically used in situations when there are uncertainties, no work that meets needs, or resources are limited [11].

The preparatory phase of Pathway 3 included three steps. The first step was establishing a core team, with team composition emerging as similar to that described for Pathway 2 (although with more emphasis on capacity for political and policy context analysis) [45, 47, 51]. The second step was defining a scope for prioritization, with our framework suggesting factors such as outer context (e.g. availability of evidence, strength of relationships among policymakers and researchers, and policymakers' capacity to use evidence) and inner context (e.g. organizational structures, resources, staffing) in which decision-making takes place as key aspects to consider, with ongoing situation analysis emerging as a tool to help refine scope [27, 38]. The final step of the preparatory phase of Pathway 3 was generating issues, with the analysis indicating that two key approaches may be used: (1) horizon scanning (where potential issues are identified and recommended for further in-depth review based on initial criteria assessment); and (2) ad hoc processes, driven by political, policy and systems analysis to understand which issues are likely to be on a governmental or decision-agenda and the likelihood that they’ll be the focus of future decision-making (e.g., by identifying windows of opportunity for action and the factors shaping the policy process) [34, 47].

The prioritization phase of Pathway 3 included two steps. The first step is selecting prioritization criteria, balancing responsiveness to emergent events with long-term strategic goals (e.g., prioritizing those issues where there is a window of opportunity to push it to the top of a government’s decision agenda), and embedding flexibility to be both proactive and reactive when needed. The second step in this phase of Pathway 3 is communicating priorities, which focuses on documentation and communication (e.g. linking issues to public agendas or international commitments, engaging champions, etc.) that can trigger mutual understanding and increase policymakers' and stakeholders' buy-in to the prioritized issues [38].

The knowledge translation phase of Pathway 3 includes two steps. The first step is determining the appropriate venue (with factors similar to knowledge translation phase of Pathway 1 identified) and the second step is implementing the priorities/KT efforts, which was also similar to those identified in this phase of Pathway 1 [11, 29, 45, 47].

The final phase—scale-up and sustainability—in Pathway 3 includes three steps: monitoring and evaluation, institutionalization and engaging in peer learning. For the first step, we found that a monitoring and evaluation plan is crucial in Pathway 3 to assess the judicious use of resources, the responsiveness of products to policy needs, and the extent to which evidence generated is utilized in decision-making [14, 29]. The details for institutionalization and peer learning, which are steps 2 and 3 in this pathway, align with those described in Pathway 1.

#### Cross-cutting constructs across Pathways 1, 2 and 3

Finally, as shown in Table 2, we also identified cross-cutting constructs across all pathways and phases, including ‘stakeholder engagement’ and ‘capacity strengthening’. Details of how these cross-cutting themes influence dimensions of the collaborative, demand-driven and supply-driven pathways are included in Table 3.

**Table 3:** Roles of stakeholder engagement and capacity strengthening across pathways and phases

Cross-cutting constructs	Description
Engaging stakeholders	Stakeholder engagement is widely advocated throughout the priority-setting process as it fosters collaboration, responsiveness, and validation, which in turn increases the relevance and impact of issues prioritized for knowledge translation. The studies highlighted the importance of engaging the right stakeholders from the start, and throughout the process [30, 36, 45, 47]. In the collaborative pathway, engagement of diverse stakeholders is essential during the preparatory and prioritization phases to define the scope, establish timelines, generate potential issues, agree on the prioritization criteria and validate a final list of prioritized issues for KT [13, 14]. This engagement continues into the knowledge translation phase, where stakeholders contribute to the design and dissemination of KT products, ensuring relevance and accessibility. Sustaining engagement with stakeholders is crucial in the final phase to monitor the impact of KT products and adapt priority setting approach for long-term policy influence and improvement [12, 13, 45]. In the demand-driven pathway, stakeholder engagement takes the form of direct interaction with policymakers or stakeholders who identify specific issues or request evidence; effective engagement involves understanding their needs and providing timely, policy-relevant KT product [29, 60]. In the supply-driven pathway, stakeholder engagement is crucial for validating and refining the identified needs or gaps and ensuring that the KT products are effectively aligned with real-world requirements [45] Several challenges have also been cited while engaging stakeholders such as their limited time availability, administrative burden, and their poor technical skills in priority-setting and knowledge translation tools among others [30, 31].
Strengthening capacity	Capacity strengthening supports the effectiveness of each pathway and phase by building the skills and resources necessary for successful priority setting. A noted challenge to effective priority setting is the limited understanding of the priority setting process and the lack of technical skills in priority setting as a knowledge translation tool [30, 31]. Capacity strengthening should ideally encompass the individual, organizational and systems level capacities [33, 34, 63, 66]. Individual-level capacity strengthening is integral to each pathway; in the collaborative pathway, building the capacity of stakeholders through training in collaborative methods, data interpretation, and priority-setting frameworks is necessary for effectively participating in and contributing to systematic priority-setting processes [32–34, 67]. In the demand-driven pathway, strengthening the capacity of policymakers and stakeholders through enhancing skills in problem identification, evidence appraisal, and decision-making can help ensure they can articulate their needs clearly, seek out relevant evidence, and apply it effectively [50]. In the supply-driven pathway, capacity strengthening focuses on equipping KT platforms to identify and address policy gaps efficiently through improving skills in needs assessment, evidence synthesis, and gap analysis. The organizational level capacities include organizational strengthening for priority setting as a KT strategy (including priority setting tools and resources; protected time for staff; structure for producer-user interface; and infrastructure and incentives to support and facilitate priority-setting initiatives) [12, 54] At the systems level, strong policy leadership support is required to promote a receptive climate for priority setting as a critical activity for promoting evidence-informed decision-making [54, 66]

### Contexual factors affecting the pathways, phases and steps associated with the prioritization of issues for knowledge translation efforts

Prioritization of issues for KT efforts is influenced by a range of contextual factors. Our findings unpacked the following four categories (see Table 4 and Additional file 2 for a detailed narrative description):

- **Institutions:** The configuration of a country's government structure and policymaking processes play a foundational role in shaping the prioritization of policy issues for KT through impacting resource allocation, attention allocation, and national agenda-setting processes. Concepts covered include government structures, health sector reforms, network structures, power of guiding organizations, and evidence support systems.
- **Interests:** This category examines the forces shaping policy issue prioritization for KT. highlighting the dynamic interplay between external pressures and internal motives guiding the policy agenda. Concepts covered include pressure from local interest groups, public preferences, external actors (e.g. donors), policy champions and entrepreneurs, policy community cohesion, and the role of knowledge brokers.
- **Ideas:** This category explores the cognitive and intellectual forces that intersect to shape the trajectory of issues on the policy agenda. They shape agenda setting and issue prioritization by determining which representations of the problem and potential solutions will be heard and understood by policymakers. Concepts covered include political will and commitment, change in political discourse, issue type, problem recognition, framing, availability of alternative policy solutions, and equity concerns.
- **External events that affect political context:** This category underscores the role of external events in shaping policy priorities. Concepts covered include changes in socioeconomic conditions, focusing events, technological advancements, and global trends.

**Table 4:** Contextual factors influencing prioritization of issues for knowledge translation efforts and mechanisms of influence

Categories	Types of factors/features	Mechanisms of influence	References
Institutions	Government structure and governance dynamics	• Set the political agenda • Shape priority-setting and resource allocation frameworks • Influence financial mobilization for issues • Constrain or incentivize choices available and so affect what issues will (or will not) emerge at the head of national agendas	[10, 22, 46, 52, 68, 69]
	Government-led health sector reforms	• Open policy windows, fostering a prime juncture for elevating certain issues • Promote accountability mechanisms which create receptive environments for evidence • Influence policy and drive policy transformation that prioritize specific issues • Stimulate demand for evidence and knowledge translation products • Set agendas and enact policies that prioritize specific issues aligned with governmental priorities or public mandates	[70]
	Established processes for decision-making	• Shape priority setting and resource allocation frameworks • Promote accountability and openness to consultative and deliberative processes • Emphasize evidence-informed decision-making and so can affect the demand for evidence and knowledge translation products	[52]
	Policy legacies	• Create a path dependency, where past decisions shape and constrain future priorities and choice	[55]
	Network structures	• Influence attention and resource allocation • Shift the dynamics of policy issue prioritization • Mobilize support around specific issues • Facilitate effective communication and collaboration • Reduce resistance and increase receptiveness to prioritize address specific issues • Collectively determine the course of issues that demand knowledge translation	[56]
	Influence of guiding organizations	• Foster policy communities and steer the agenda-setting process • Exert pressure and drive collective action • Set the tone for specific issues, thereby generating political momentum	[71–75]
	Evidence systems	• Foster a supportive climate for evidence use • Stimulate demand for evidence and knowledge translation • Facilitate knowledge translation and evidence-based approaches • Promote evidence-driven policy decisions	[10, 50, 52, 66, 76]
**Interests**	Interest group pressure	• Influence the agenda-setting process • Form alliances and networks to collectively exert pressure to prioritize certain policy issues over others • Garner public attention for specific issues • Mobilize support and facilitate collaborations that amplify their influence in setting KT priorities	[66, 68, 77–81]
	Citizens and public preferences	• Bring attention to certain issues through raising awareness, mobilizing support, and advocating for specific issues • Shape the national mood and policy directions • Exert pressure on governments to address specific issues • Raise awareness and galvanize public support for pressing issues	[70, 82]
	Influence of external actors (i.e. funders, donors and influence of globally recognized positions of authority)	• Influence attention, reshape policy trajectories and resource allocation and shift overall power dynamics • Affect the composition and structure of actors in the networks, which enable the entry and dissemination of new ideas • Mobilize support and facilitate collaborations that amplify their influence in setting KT priorities • Direct financial and human resources towards prioritized KT initiatives	[69, 74]
	Policy champions and entrepreneurs	• Shape the discourse and influence how policymakers and the public perceive the importance and urgency of issues • Create a stronger collective voice to push for the prioritization of specific issues • Leverage networks to connect with key decision-makers, experts, and stakeholders who have the power to influence the prioritization of policy issues • Provide political support by endorsing specific policies • Influence policy and drive policy transformation	[68, 79, 81, 83–86]
	Policy community cohesion	• Foster collaboration, shared understanding, and effective communication within the policy-making environment • Achieve a uniform voice and a common ground of values • Facilitate the identification and prioritization of policy issues that are collectively considered important • Create a groundswell of support, making it more likely that knowledge translation products will be well-received and utilized	[68, 77–79, 83, 84, 86–88]
	Role of knowledge brokers	• Advocate for evidence-based solutions, influencing decision-makers to prioritize topics that promise significant scientific or societal impact • Advocate for particular priority setting and resource allocation frameworks • Advocate for certain policy issues by effectively communicating the importance of specific research findings • Bring attention to specific issues, helping to shape the agenda for policymakers • Monitor the policy environment, identifying emerging issues or changes in priorities • Contribute to the development of knowledge translation products that are well-informed, relevant, and persuasive	[10, 64, 66, 71, 82]
**Ideas**	Political will and commitment	• Shape the prioritization and advancement of policy issues • Influence lever of attention and resources for an issue • Garner support for an issue • Create demand for evidence and knowledge translation	[58, 72, 73, 80, 83, 84, 87, 89]
	Alignment with national agendas and goals	• Enhance the political and public support for policy issues • Guide resource allocation • Provide a strategic focus for knowledge translation efforts	[70, 73, 84, 87, 88]
	Issue type (contestability versus saliency of issue)	• Influence level of consensus on an issue • Influence lever of attention and resources for an issue • Influence demand for evidence and knowledge translation	[17, 34, 39, 67, 70, 80, 82, 83, 89–92]
	Problem recognition	• Shape discussions and frame issues • Set priorities by highlighting the severity and magnitude of different problems • Present evidence, potential consequences of inaction, and possible solutions for an issue • Prompt a need for evidence-based solutions • Provide a strategic focus for knowledge translation efforts	[84, 90, 93]
	Framing	• Employ social, psychological and cultural concepts to imbue issues with meaning and interpretation • Shape how policymakers perceive the nature and causes of an issue • Influence how important an issue is perceived to be • Make certain aspects of an issue more salient and relevant • Align the issue with the values of policymakers or the public, leading to increased attention and prioritization • Build broader support for prioritization	[25, 39, 63, 66, 69, 72, 85, 94]
	Availability of alternative policy options	• Reduce resistance and increase receptiveness to prioritize address the issue • Facilitate knowledge translation and evidence utilization	[25, 73, 80, 83, 89, 90]
	Shifts in political discourse	• Influence the public agenda and policymakers' priorities • Shape how issues are framed and perceived by the public and policymaker • Influence the language and narratives used in policy discussions • Emphasize certain policy goals or objectives, prompting a re-evaluation of priorities	[95]
	Equity and health concerns	• Identify areas where health disparities exist, which can highlight priority issues that require attention • Stimulate demand for evidence and knowledge translation	[71, 93]
**External events that affect political context**	Changes in socioeconomic conditions	• Give rise to new challenges or opportunities • Exacerbate or mitigate existing challenges and inequalities • Shift the policy agenda, making certain issues more pressing or redirecting attention to previously overlooked areas • Influence public opinion and political will	[83, 85, 91, 92]
	Focusing events	• Create sense of urgency and severity • Capture public interest and concern • Bring specific issues to the forefront of public and policymaker attention • Influence resource allocation and financial mobilization • Foster policy innovation • Stimulate demand for evidence and knowledge translation	[63, 67, 70, 80, 83, 91, 92]
	Geopolitical developments	• Reshape the policy landscape, altering the relevance and importance of various issues • Lead to shifts in policy direction • Stimulate demand for evidence and knowledge translation	[47]
	Technological advancements and innovations	• Highlight gaps in current healthcare systems and practices, prompting a re-evaluation of priorities • Open policy windows and drive policy transformation • Stimulate demand for evidence and knowledge translation	[96]
	Global trends	• Open policy windows • Highlight pressing issues and prompt a re-evaluation of priorities • Create political pressure to conform with international agreements, treaties or commitments	[25, 87, 90]

These contextual factors can come together to push an issue to be prioritized and can also influence the pathways for prioritization of issues and the development of KT products. Notably, the priority-setting process for KT efforts unfolds within a broader context of the agenda-setting process in the policymaking cycle, which is constantly evolving (i.e., of the issues prioritized by government decision-makers at any given point in time, only some will end up also being prioritized for KT efforts).

## Discussion

### Principal findings and interpretations

This CIS resulted in a preliminary multi-level conceptual framework for KT priority-setting, filling an important conceptual gap in the literature and providing systematic and granular guidance to stakeholders on health-focused priority-setting efforts for KT and EIP.

Our findings reveal three main pathways for identifying and prioritizing issues for KT efforts that are ultimately meant to respond to decision-makers’ needs: (1) collaborative, involving policymakers and stakeholders in an explicit and structured process; (2) demand-driven, where policymakers or stakeholders request evidence on specific issues; and (3) supply-driven, where KT platforms identify a need or policy gap. These three pathways reflect the reality that policymaking is rarely linear, and that KT efforts must align with institutional readiness, available capacity, and political context offering flexible entry points depending on system maturity, urgency, and governance dynamics. Thus, making the conceptual framework adaptable across diverse policy settings. They also challenge the status quo which assumes a reactive approach for targeted KT efforts, where KT practitioners wait for the policy process itself to determine what they should focus on, rather than expect they can engage in a rational, linear-type process with policymakers to determine which issues are front-and-center and could benefit from targeted KT efforts.

Within each pathway, we identified four interconnected phases depicting the process of priority setting: “Preparatory”, “Prioritization”, “Knowledge Translation” and “Scale-up and Sustainability”. The four phases are interconnected and the steps that take place in one phase can influence others. In the supply and demand-driven pathways, the preparatory and prioritization phases could potentially be integrated as opposed to being presented as distinct phases. The KT phase was less emphasized where capacity or skills were lacking. Evidence on ‘Scale-up and Sustainability’ phase was relatively limited, albeit the included documents underscore its importance in embedding priority setting within KT and decision-making processes. A significant contribution of the framework is the explicit and systematic integration of stakeholder engagement and capacity strengthening as cross-cutting elements. Unlike many existing frameworks where these components are referenced only tangentially, our framework embeds them throughout all phases and pathways, thereby enhancing coherence, inclusivity, and potential for sustained impact.

While many frameworks identified in the literature remain theoretical and make abstraction of the country contexts, our framework explicitly considers contextual factors, making it more agile, adaptable and policy-relevant. We identified four key contextual categories—institutions, ideas, interests and external events—that can influence the three pathways for KT prioritization and the development of KT products, and thus need to be integrated into the design and implementation of priority-setting processes. The most frequently observed contextual factors across these categories include: problem recognition (indicator, report, focusing events), stakeholder interest, issue type (e.g., salient versus contestable), political commitment and alignment with national goals and agendas. Our analysis also indicates that priority-setting for KT efforts should not occur in isolation from the broader agenda-setting process in policymaking where issues are prioritized for policy attention and action. Rather, KT prioritization unfolds within and alongside this broader agenda-setting stage, addressing only a subset of those policy issues.

In conclusion, the findings informing the suggested framework’s structure—encompassing three prioritization pathways, four process phases, and four contextual categories—offer both strategic direction and operational clarity to stakeholders designing KT interventions. While there is no single optimal pathway for prioritizing issues for KT products, our findings suggest that priority setting for EIP should balance the need for rapid and efficient processes with the need for processes that are explicit, systematic and fair [11, 29, 45, 47, 63]. This approach would need to be dynamic and revised every few weeks or months to maintain a meaningful balance between proactive and reactive priorities. We found limited evidence on the link between contextual factors and the different pathways [64] (see Table 5), highlighting the need for further research (e.g. through robust case-studies of KT prioritization processes) to explore and unpack these intersections and relationships.

**Table 5:** Insights on potential links between contextual factors and priority-setting pathways

Contextual categories	Link to priority-setting pathways			Implications
	Collaborative	Demand-driven	Supply-driven	
Institutions	Culture and structure of institutions can either facilitate or hinder collaboration. Supportive institutional frameworks encourage the involvement of diverse stakeholders in priority setting processes.	Institutions need to be responsive to the needs of end-users and should have mechanisms in place to identify and prioritize those needs. Receptive environment for evidence use encourages evidence-driven policy decisions and subsequent demand for evidence.	Knowledge translation institutions influence the types of research prioritized based on their expertise and strategic goals.	• Understand the organizational structures, cultures, and policies that may influence priority setting. Foster collaboration and communication among different institutions • Establish clear channels for information exchange and decision-making • Address institutional barriers that may hinder prioritization process
Ideas	Diverse ideas are welcomed and integrated into the collaborative process. The focus is on incorporating a broad range of perspectives to collectively shape the policy agenda for knowledge translation.	Ideas emerge from the identified needs of end-users, shaping the knowledge translation agenda to address practical issues.	The prioritization of ideas is often driven by the intellectual curiosity and expertise of knowledge brokers within the institution.	• Foster an environment that encourages creativity and innovation • Consider input from a variety of sources, including researchers, practitioners, and the communities affected by the issue • Balance competing interests to ensure that the prioritization process is fair and aligns with the broader objectives of knowledge translation
Interests	Balancing conflicting interests among stakeholders is crucial for consensus. Transparency in decision-making helps manage interests effectively.	End-user interests guide agenda for knowledge translation. Identifying and aligning interests with practical outcomes is key.	Knowledge broker academic interests and the institution's strategic goals influence the prioritization of issues for knowledge translation products.	• Develop transparent and inclusive processes for identifying and reconciling conflicting interests • Strive for a balance that aligns with the overall goals of knowledge translation
External events	External events can trigger the need for collaboration, as issues may arise that require a collective response.	External events may highlight new challenges or emphasize the urgency of existing needs, influencing priorities for knowledge translation.	External events can stimulate knowledge brokers to explore emerging issues, aligning their work with current trends and demands.	• Build flexibility into the priority setting process to adapt to unforeseen external events • Establish mechanisms for ongoing monitoring and evaluation to ensure that priorities remain relevant • Foster a culture of agility and adaptability within the knowledge translation framework

### Implications for policy and research

Priority setting in KT is often not undertaken in a systematic, transparent, or inclusive manner. Rather, decisions about which policy issues to pursue are frequently reactive, shaped by immediate demands, individual preferences, or ad hoc requests. Such fragmented practices risk overlooking critical issues, misaligning efforts with national priorities, misallocating limited resources, and undermining the legitimacy and effectiveness of KT initiatives. In light of the potential consequences of these unsystematic approaches, there has been a growing call, particularly from stakeholders such as EVIPNet teams, for more structured and methodologically rigorous priority-setting processes. This shift reflects an increasing recognition that systematic approaches are fundamental to enhancing the relevance, equity, and impact of evidence-informed policymaking.

The different pathways and phases, as well as the contextual factors presented in the proposed framework, offer a deeper understanding of priority-setting as a KT strategy, and initial guidance to countries and stakeholders in designing and implementing priority-setting for impactful KT efforts. Country-specific needs and readiness should be carefully considered to determine what is required in each phase and which pathway would be the best starting point for a given context. Importantly, rather than striving for a single way of prioritizing issues for KT products, our findings suggest that policymakers and KT practitioners have to combine a proactive approach to priority-setting that contributes to future plans (e.g., what priority should an issue be given in a national health strategic plan) with a reactive approach that is responsive to emerging needs, unanticipated events and opportunities (i.e., time-sensitive). All three pathways should ideally be considered to achieve optimal impact at the many different stages and multiple ‘ways in’ to the decision-making process. An overarching leadership and governance structure could enhance this process by designing a flexible, adaptable priority-setting approach that can be applied to different issues and levels, operationalizing and balancing the different pathways for priority setting, providing guidance on research, political and policy contexts, and overseeing the overall implementation.

Moreover, while decision-makers and KT practitioners are not oblivious to politics and its effects, the particular influence of institutions, interests, ideas, and external factors on priority-setting and resource allocation often remain overlooked [65]. Explicitly acknowledging these factors enhances the adaptability of priority-setting processes. Understanding stakeholders' interests and their influence through various mechanisms (e.g. resource allocation, political influence, specialized expertise, organizational positioning, and advocacy efforts) is particularly crucial.

Our findings also have implications for WHO work. The proposed framework offers a solid foundation for the development of a priority-setting tool and guiding manual supporting countries in designing impactful KT products addressing the most pressing policy questions. Additionally, findings can be integrated into existing WHO reference documents on KT, specifically WHO EVIPNet Evidence Briefs for Policy manual [45].

Future initiatives could use the framework to guide prioritization of issues for KT products and EIP and assess its applicability. Researchers and KT practitioners might further refine and test the framework in different regions and systems, explore specific framework elements, and unpack them for added precision and clarity, drawing from various fields of scholarship. Future work could also clarify relationships between the pathways, steps, and contextual factors, and explore effective methods for institutionalizing priority-setting for KT and EIP.

There could also be broader implications for KT work beyond the health sector. The framework’s core elements—pathways, phases, contextual integration—can be adapted for use in other domains such as education, climate policy, or social protection. In volatile contexts, such as public health emergencies or humanitarian crises, layering this framework with real-time monitoring systems can ensure responsiveness and relevance.

### Strengths and limitations

This study represents one of the first attempts to build a more comprehensive understanding of priority-setting for KT efforts. While previous studies have focused on research priority-setting, often framed within the construct of addressing knowledge gaps as part of broader KT efforts, this study specifically examines how KT products (e.g., evidence briefs and rapid syntheses)—ultimately meant to respond to decision-makers’ needs—are prioritized. On the whole, the emerging framework and theoretical propositions developed here constitute a significant first attempt to understand a very complex field of inquiry. The CIS methodology allowed for a rich, theory-informed synthesis of diverse body of literature. It encompassed a broad interdisciplinary search across domains and topics, integrating insights from political science and policy analysis, and both conceptual and empirical studies. The iterative sampling approach, combined with the inclusion of both empirical and conceptual studies, strengthens the methodological rigour and enhances the robustness and credibility of the findings.

Potential limitations include the under-representation of the literature from public policy and social sector as the search terms yielded limited results from those fields. Additionally, despite efforts to include theoretical papers, the majority of the reviewed literature was empirical. To address these limitations, we consulted experts in the field and searched for specific concepts needed to fill the conceptual gaps. Moreover, while the initial electronic database search took place in February 2022, two additional rounds of purposive sampling were conducted in June 2022 and May 2023 to help with the interpretive process leading to the conceptual framework. We stopped sampling at the saturation point. This sampling approach ensured that relevant published literature beyond the initial search date was captured.

## Conclusion

This CIS generated a multi-level conceptual framework for prioritizing policy issues for KT efforts, contributing meaningfully to both the literature and the operationalization of KT and EIP. The framework outlines three pathways for identifying and prioritizing issues, four process phases, and four contextual categories that may influence prioritization. It offers both strategic direction and operational clarity to stakeholders engaged in health-focused priority-setting for KT and EIP, and should be carefully aligned with country-specific needs and readiness to maximize its potential for sustained impact.

## Supplementary Information

Additional file1 (PDF 83 KB)

Additional file2 (PDF 228 KB)

## Data Availability

All data generated or analyzed during this study are included in this published article and its supplementary information files.

## Abbreviations

CIS: Critical interpretive synthesis
EIP: Evidence-informed policymaking
KT: Knowledge translation
