# Supplementary material for: Prioritizing policy issues for knowledge translation: a critical interpretive synthesis
Source: Glob Health Res Policy. 2025 Aug 20;10:35. doi: 10.1186/s41256-025-00440-y (PMC12366224; doi:10.1186/s41256-025-00440-y)
Supplement: Supplementary file 1 — Additional file1 (PDF 83 KB) [file 41256_2025_440_MOESM1_ESM.pdf]

## Appendix 1: Search strategies for the different databases

### Database: Ovid MEDLINE(R) and In-Process, In-Data-Review & Other Non-Indexed Citations <1946 to February 03, 2022>

Search Strategy:

--------------------------------------------------------------------------------

1 health planning guidelines/ or health planning technical assistance/ or health priorities/ or regional health planning/ (20722)

2 health care rationing/ (11969)

3 resource allocation/ (9075)

4 ("health planning" or "health care rationing").tw. (3729)

5 (priorit* adj3 (set or setting or rank* or identif*)).ti,ab. (12969)

6 (agenda? adj2 (setting or shaping)).ti,ab. (760)

7 (resource? adj1 allocati*).ti,ab. (10731)

8 1 or 2 or 3 or 4 or 5 or 6 or 7 (61087)

9 Public Policy/ or policy making/ or health policy/ or government programs/ or health systems plans/ (116984)

10 (policymaking or policy-making or policy-maker? or decision-maker?).ti,ab. (47163)

11 (health adj system? adj plan?).ti,ab. (28)

12 (Government* adj1 (policy or policies or program? or strategy or strategies or plan? or regulation? or decision*)).ti,ab. (6954)

13 (policy adj (maker? or making)).ti,ab. (29760)

14 (policy adj2 (topic? or issue? or problem or option?)).ti,ab. (5084)

15 Translational Medical Research/ (12133)

16 ((knowledge or evidence or research) adj2 (exchang* or transfer* or translati* or brokering or uptake or synthes* or utilization)).tw. (40725)

17 ((knowledge or evidence or research) adj ("use" or implementation or disseminat*)).ti,ab. (3118)

18 ((deliberative or policy or stakeholder?) adj2 dialogue?).ti,ab. (428)

19 ((evidence or policy) adj (brief? or summar*)).ti,ab. (1016)

20 "rapid response".ti,ab. (6762)

21 9 or 10 or 11 or 12 or 13 or 14 or 15 or 16 or 17 or 18 or 19 or 20 (220888)

22 ((Priority? or priorities or prioritizing or prioritization?) adj1 health adj1 (topic? or issue? or problem?)).ti,ab. (204)

23 ((Priority? or priorities or prioritizing or prioritization?) adj2 (policy* or policies or politic*)).ti,ab. (1625)

24 8 and 21 (9726)

**Database: Embase**

……………………………………………………

| No. Query | Results |
| --- | --- |
| #25. #24 AND (2000:py OR 2001:py OR 2002:py OR 2003:py OR 2004:py OR 2005:py OR 2006:py OR 2007:py OR 2008:py OR 2009:py OR 2010:py OR 2011:py OR 2012:py OR 2013:py OR 2014:py OR 2015:py OR 2016:py OR 2017:py OR 2018:py OR 2019:py OR 2020:py OR 2021:py) | 4,183 |
| #24. #19 AND #23 | 4,678 |
| #23. #17 OR #18 OR #22 | 7,179 |
| #22. #20 AND #21 | 5,891 |
| #21. #6 OR #7 OR #8 OR #9 OR #10 OR #11 OR #12 OR #13 OR #14 OR #15 OR #16 | 287,818 |
| #20. #1 OR #2 OR #3 OR #4 OR #5 | 44,735 |
| #19. framework:ab,ti OR frameworks:ab,ti OR model:ab,ti OR models:ab,ti OR approach:ab,ti OR approaches:ab,ti OR tool:ab,ti OR tools:ab,ti OR checklist:ab,ti OR checklists:ab,ti OR strategy:ab,ti OR strategies:ab,ti OR process:ab,ti OR processes:ab,ti OR criteria:ab,ti OR toolkit:ab,ti OR technique:ab,ti OR techniques:ab,ti OR method:ab,ti OR methods:ab,ti OR theory:ab,ti OR theories:ab,ti OR exercise?:ab,ti | 17,084,640 |
| #18. ((priority? OR priorities OR prioritizing OR prioritization?) NEAR/2 (policy* OR policies OR politic*)):ab,ti | 943 |
| #17. ((priority? OR priorities OR prioritizing OR prioritization?) NEAR/2 (topic? OR issue? OR problem?)):ab,ti | 426 |
| #16. 'rapid response':ab,ti | 9,157 |
| #15. ((evidence OR policy) NEXT/1 (brief? OR summar*)):ab,ti | 959 |
| #14. ((deliberative OR policy OR stakeholder?) NEXT/2 dialogue?):ab,ti | 79 |
| #13. ((knowledge OR evidence OR research) NEXT/1 ('use' OR implementation OR disseminat*)):ab,ti | 4,692 |
| #12. ((knowledge OR evidence OR research) NEAR/2 (exchang* OR transfer* OR translati* OR brokering OR uptake OR synthes* OR utilization)):ab,ti | 53,602 |
| #11. 'knowledge translation'/de | 78 |
| #10. (policy NEXT/2 (topic? OR issue? OR problem OR option?)):ab,ti | 4,453 |
| #9. 'policy maker'/de | 16 |
| #8. 'policy maker':ab,ti OR 'policy making':ab,ti | 6,555 |

| | |
|---|---|
| #7. (government* NEXT/1 (policy OR policies OR program? OR strategy OR strategies OR plan? OR regulation? OR decision*)):ab,ti | 6,638 |
| #6. 'health care policy'/de OR 'public policy'/de | 210,427 |
| #5. (resource? NEXT/1 allocati*):ab,ti | 493 |
| #4. (agenda* NEAR/2 (setting OR set OR shaping)):ab,ti | 1,208 |
| #3. (priorit* NEAR/3 (set OR setting OR rank* OR identif*)):ab,ti | 16,667 |
| #2. 'health planning':ab,ti OR 'health care rationing':ab,ti | 4,749 |
| #1. 'resource allocation'/exp | 22,482 |

…………………………………………………

**Database:CINAHL** **Interface - EBSCOhost Research Databases**

| # | Query | Limiters/Expanders | Results |
|---|---|---|---|
| S21 | S7 AND S20 | Expanders - Apply equivalent subjects<br>Search modes - Boolean/Phrase | 2,428 |
| S20 | S8 OR S9 OR S10 OR S11 OR S12 OR S13 OR S14 OR S15 OR S16 OR S17 OR S18 OR S19 | Expanders - Apply equivalent subjects<br>Search modes - Boolean/Phrase | 180,169 |
| S19 | TI "rapid response" OR AB "rapid response" OR SU "rapid response" | Expanders - Apply equivalent subjects<br>Search modes - Boolean/Phrase | 2,503 |
| S18 | TI ( (evidence or policy) N1(brief? or summar*) ) OR AB ( (evidence or policy) N1(brief? or summar*) ) OR SU ( (evidence or policy) N1(brief? or summar*) ) | Expanders - Apply equivalent subjects<br>Search modes - Boolean/Phrase | 4,495 |
| S17 | TI ( (deliberative or policy or stakeholder?) N2 dialogue? ) OR AB ( (deliberative or policy or stakeholder?) N2 dialogue? ) OR SU ( (deliberative or policy or stakeholder?) N2 dialogue? ) | Expanders - Apply equivalent subjects<br>Search modes - Boolean/Phrase | 318 |
| S16 | TI ( (knowledge or evidence or research) N1 ("use" or implementation or disseminat*) ) OR AB ( (knowledge or evidence or research) N1 ("use" or implementation or disseminat*) ) | Expanders - Apply equivalent subjects<br>Search modes - Boolean/Phrase | 17,330 |
| S15 | TI ( (knowledge or evidence or research) N2 (exchang* or transfer* or translati* or brokering or uptake or synthes* or utilization) ) OR AB ( (knowledge or evidence or research) N2 (exchang* or transfer* or translati* or brokering or uptake or synthes* or utilization) ) OR SU ( (knowledge or evidence or research) N2 (exchang* or transfer* or translati* or brokering or uptake or synthes* or utilization) ) | Expanders - Apply equivalent subjects<br>Search modes - Boolean/Phrase | 23,887 |
| S14 | (MH "Translational Medical Research") OR (MH "Knowledge Management") | Expanders - Apply equivalent subjects<br>Search modes - Boolean/Phrase | 2,364 |
| S13 | TI ( policy N2 (topic? or issue? or problem or option?) ) OR AB ( policy N2 (topic? or issue? or problem or option?) ) OR SU ( policy N2 (topic? or issue? or problem or option?) ) | Expanders - Apply equivalent subjects<br>Search modes - Boolean/Phrase | 4,520 |

| | | | |
|---|---|---|---|
| S12 | TI ( policy N1 (maker? or making) ) OR AB ( policy N1 (maker? or making) ) OR SU ( policy N1 (maker? or making) ) | Expanders - Apply equivalent subjects<br>Search modes - Boolean/Phrase | 30,705 |
| S11 | TI ( Government* N1 (policy or policies or program? or strategy or strategies or plan? or regulation? or decision*) ) OR AB ( Government* N1 (policy or policies or program? or strategy or strategies or plan? or regulation? or decision*) ) OR SU ( Government* N1 (policy or policies or program? or strategy or strategies or plan? or regulation? or decision*) ) | Expanders - Apply equivalent subjects<br>Search modes - Boolean/Phrase | 30,766 |
| S10 | TI health N1 system? N1 plan? OR AB health N1 system? N1 plan? OR SU health N1 system? N1 plan? | Expanders - Apply equivalent subjects<br>Search modes - Boolean/Phrase | 136 |
| S9 | TI ( policymaking or policy-making or policy-maker? or decision-maker? ) OR AB ( policymaking or policy-making or policy-maker? or decision-maker? ) OR SU ( policymaking or policy-making or policy-maker? or decision-maker? ) | Expanders - Apply equivalent subjects<br>Search modes - Boolean/Phrase | 36,682 |
| S8 | (MH "Public Policy") OR (MH "Policy Making") OR (MH "Health Policy") | Expanders - Apply equivalent subjects<br>Search modes - Boolean/Phrase | 89,445 |
| S7 | S1 OR S2 OR S3 OR S4 OR S5 | Expanders - Apply equivalent subjects<br>Search modes - Boolean/Phrase | 11,792 |
| S6 | TI resource? N1 allocati* OR AB resource? N1 allocati* OR SU resource? N1 allocati* | Expanders - Apply equivalent subjects<br>Search modes - Boolean/Phrase | 16,189 |
| S5 | TI ( agenda? N2 (setting or shaping) ) OR AB ( agenda? N2 (setting or shaping) ) OR SU ( agenda? N2 (setting or shaping) ) | Expanders - Apply equivalent subjects<br>Search modes - Boolean/Phrase | 681 |
| S4 | TI ( priorit* N3 (set or setting or rank* or identif*) ) OR AB ( priorit* N3 (set or setting or rank* or identif*) ) OR SU ( priorit* N3 (set or setting or rank* or identif*) ) | Expanders - Apply equivalent subjects<br>Search modes - Boolean/Phrase | 6,753 |
| S3 | TI ( "health planning" or "health care rationing" ) OR AB ( "health planning" or "health care rationing" ) OR SU ( "health planning" or "health care rationing" ) | Expanders - Apply equivalent subjects<br>Search modes - Boolean/Phrase | 1,194 |
| S2 | (MH "Resource Allocation") | Expanders - Apply equivalent subjects<br>Search modes - Boolean/Phrase | 3,077 |
| S1 | (MH "Health Priorities") | Expanders - Apply equivalent subjects<br>Search modes - Boolean/Phrase | 359 |

**Database** (MAINSUBJECT.EXACT("Priorities") OR (ab(priorit* NEAR/2 (set OR setting OR rank* OR identif*)) OR ti(priorit* NEAR/2 (set OR setting OR rank* OR identif*))) OR (ab("health planning" OR "health care rationing") OR ti("health planning" OR "health care rationing")) OR (ab(agenda?

NEAR/2 (setting OR shaping)) OR ti(agenda? NEAR/2 (setting OR shaping))) OR (ab(resource? NEAR/1 allocati*) OR ti(resource? NEAR/1 allocati*))) AND ((MAINSUBJECT.EXACT("Health care policy") OR MAINSUBJECT.EXACT("Policy making") OR MAINSUBJECT.EXACT("Public policy")) OR (ab(policymaking OR policy-making OR policy-maker? OR decision-maker?) OR ti(policymaking OR policy-making OR policy-maker? OR decision-maker?)) OR (ab(policy NEAR/2 (topic? OR issue? OR problem OR option?)) OR ti(policy NEAR/2 (topic? OR issue? OR problem OR option?))) OR (MAINSUBJECT.EXACT("Knowledge management") OR MAINSUBJECT.EXACT("Knowledge sharing")) OR (ab((knowledge OR evidence OR research) NEAR/2 (exchang* OR transfer* OR translati* OR brokering OR uptake OR synthes* OR utilization)) OR ti((knowledge OR evidence OR research) NEAR/2 (exchang* OR transfer* OR translati* OR brokering OR uptake OR synthes* OR utilization))) OR (ab((knowledge OR evidence OR research) NEAR/1 ("use" OR implementation OR disseminat*)) OR ti((knowledge OR evidence OR research) NEAR/1 ("use" OR implementation OR disseminat*))) OR (ab((deliberative OR policy OR stakeholder?) NEAR/2 dialogue?) OR ti((deliberative OR policy OR stakeholder?) NEAR/2 dialogue?)) OR (ab((evidence OR policy) NEAR/1 (brief? OR summar*)) OR ti((evidence OR policy) NEAR/1 (brief? OR summar*))) OR (ab("rapid response") OR ti("rapid response")))
