# Supplementary material for: Prioritizing policy issues for knowledge translation: a critical interpretive synthesis
Source: Glob Health Res Policy. 2025 Aug 20;10:35. doi: 10.1186/s41256-025-00440-y (PMC12366224; doi:10.1186/s41256-025-00440-y)
Supplement: Supplementary file 2 — Additional file2 (PDF 228 KB) [file 41256_2025_440_MOESM2_ESM.pdf]

Appendix 2: Detailed narrative description of contextual factors influencing prioritization of issues for knowledge translation

1. Institutions

Institutions play a foundational role in shaping the prioritization of policy issues for knowledge translation. The configuration of a country's government structure, whether federal or unitary, parliamentary or presidential, lays the groundwork for the issue prioritization process. Governance dynamics, encompassing electoral processes, governmental branches, and legal mandates, exerts significant influence. Additionally, interactions between institutions, be it through partnerships, consortia, or cooperative initiatives, intricately shape the trajectory of issue prioritization, influencing resource allocation and attention allocation.

- **Government Structure and Governance Dynamics:** This factor encompasses the configuration of a country's political system (e.g., federal vs. unitary state, and parliamentary vs. presidential system) (1-5), political dynamics (shifts in power, elections, transitions in governance, and shifts in ruling parties) (6, 7) government institutional framework (governmental partnership agreements, legal jurisdictions and accountability mechanisms) (2, 3), as well as budgetary availability (resource availability and funding formulas) (1, 8, 9). These elements shape the decision-making process and ultimately determine which topics/issues receive priority on national agendas (1, 4, 8). The composition of government agencies and ministries further shapes how concerns and choices are managed across domains (10). For instance, the historical influence of dictatorship in Chile fostered a culture averse to dissent, hindering the implementation of crucial appeals processes (4). Political changes can either facilitate or hinder policy implementation (6) as observed during the transition to the George H.W. Bush administration in the U.S. The new political climate resistant to change complicated policy execution in the case of ergonomics standards (6). Additionally, forces such as budgetary and resource availability constrain or incentivize the choices available and so affect what will or will not emerge at the head of any list of priorities (1, 19). As seen in Ghana, where the 1963 Free Antenatal Care in the Public Sector Directive faced implementation challenges due to lack of resource availability (8).
- **Government-led Health Sector Reforms:** Government-led health sector reforms can trigger a transformative shift in established healthcare frameworks, fostering a prime juncture for elevating crucial topics (11, 12). These reform-driven shifts often necessitate reconfigurations of administrative and technical setups, catalyzing demand for evidence to guide policy adaptations. and accountability mechanisms through priority-setting processes (11). The mechanism of influence lies in their capacity to stimulate demand for evidence-driven policy revisions, open policy windows and drive policy transformation. For instance, substantial health system reforms in Ontario propelled the prominence of hospital-to-home transition policies, while a recent restructuring of the Ministry of Health in Canada, encompassing long-term care, provided an opportune moment to enhance the implementation of targeted interventions (11).
- **Established Processes for Decision-making:** The processes, procedures and rules set for decision-making within government bodies influence how issues are prioritized (2, 3). Institutional processes that incentivize or mandate evidence-informed decision-making can catalyze the demand for evidence and drive the creation of knowledge translation products tailored to priority concerns (6). They can also shape particular priority setting and resource allocation frameworks. Two important aspects seem crucial enablers of systematic priority-setting processes, which are the openness to consultative and deliberative processes as well as the mandate and accountability relations between the government and its agencies (2, 13).

- **Policy Legacies:** This refer to the historical policies and decisions that have been made and continue to influence the current policy landscape. These legacies create a path dependency, where past decisions shape and constrain future priorities and choices. Successes and failures in the past may influence the willingness to address similar issues in the present. Similarly, previously chosen policy responses are likely to be prioritized over alternative evidence-informed option. A study of WHO's management of the 2009 H1N1 pandemic suggested that previously chosen policy responses are likely to be prioritized over alternative evidence-informed options, especially when the health issue is surrounded by scientific uncertainties (14).
- **Network Structures:** The existence of policy networks that unite the government with actors outside of the formal process of government can also shape policy developments and choices (15-17). This extends beyond institutions to encompass their relationships, interactive patterns, and alignment with political norms and regulations, collectively shaping the political landscape. The interplay between institutions, whether through partnerships, consortia, or cooperative initiatives, goes on to shape the very trajectory of topic prioritization by influencing attention and resource allocation. Through these connections, institutions become nodes in a network, dynamically influencing one another and collectively determining the course of issues that demand knowledge translation (15-17). For instance, the Dutch governance model – where the state relies heavily on social networks to govern, exemplifies a networked approach to policy, where the state operates as one of many actors within a broader social network. Decision-making in this context is characterized by a lack of strong permanent state bureaucracy and is typically driven by broad consensus among various stakeholders (17).
- **Influence of Guiding Organizations:** Guiding organizations, — i.e. organizations or coordinating mechanisms with a mandate to lead the initiative—are also crucial in steering the prioritization of topics in various fields (18-24). Strong guiding institutions. These institutions not only catalyze demand for action but also spearhead policy initiatives, foster policy communities and steer the agenda-setting process. Notably, international entities like OECD, WHO, and UN agencies wield considerable influence by setting the tone for specific issues, thereby generating political momentum and prompting governments to address prioritized concerns (25, 26). The evolution of these initiatives typically commences within informal associations or as projects within established organizations; but their sustainability and impact hinge on their ability to evolve into self-standing, enduring institutions (27). However, the competition among organizations for dominance can hinder the establishment of such effective guiding structures (27). A notable example of a successful guiding institution is the Task Force for Child Survival and Development, led by Grant, which has made significant strides in child health advocacy and policy influence, demonstrating the potential impact of well-structured guiding bodies in shaping topic prioritization.
- **Evidence Systems**: The importance attached to the use of evidence is a crucial enabler of priority setting processes (1). This involves how organizations, rules, and procedures are designed to encourage or hinder the use of evidence in decision-making processes. A strong evidence system facilitates evidence-driven policy decisions and subsequent demand for evidence while an unreceptive one hinders evidence-based approaches. Mechanisms to foster a supportive climate include enhancing capabilities, addressing attitudes towards evidence, and altering institutional structures to strengthen evidence use (6, 28-30). In Malawi, the integration of House KTPMalawi within the Ministry of Health's research department significantly enhanced the capacity, demand, and support for using evidence in health policy-making, reflecting a receptive climate for evidence utilization (30).

2. Interests:

The "Interests" category encompasses an array of influential forces shaping the prioritization of policy issues for knowledge translation. These forces underscore the dynamic interplay between different group pressures and internal motives guiding the policy agenda. Factors under this category include pressure from local interest groups, influence of external actors, policy champions, level of cohesion, and the role of knowledge brokers.

- **Interest Group Pressure:** Local interest groups, including media, civil society, and professional associations, hold the power to magnify specific issues on the policy radar (25, 26, 31-41). Shifts in the interests of different groups within society, including changes in public sentiment or the priorities of key interest groups, could impact the prioritization of policy issues. For example, in Sri Lanka, grassroots organizations, although perceived as marginal political actors by the government, effectively mobilized to pressurize political leaders to address violence against women (41). This underscores the potential impact of civil society movements in altering policy agendas, even in the face of governmental reluctance or indifference. While in Lebanon, media coverage highlighting medical errors spurred a knowledge translation center to develop policy briefs, demonstrating the media's role in shaping public discussions and influencing policy responses (42).
- **Citizens and Public Preferences:** The influence of the public, encompassing patients, families, and caregivers, in directing the prioritization of topics cannot be overstated. Their ability to exert pressure on governments and articulate their needs plays a crucial role in agenda-setting and policy formulation (11, 43, 44). This grassroots influence is a testament to the power of citizen engagement in democratic processes, where public opinion and preferences significantly shape the national mood and policy directions (45). For instance, in Canada, the priorities expressed by patients, families, and caregivers, such as the need for improved hospital-to-home transitions, have directed attention and resources towards addressing these specific healthcare challenges (11). Moreover, the growing public awareness of health issues can create a palpable pressure on governments to respond. A pertinent example of this is the public's increasing awareness of the negative health impacts of trans fats in food products across Europe (44). This heightened awareness has led to an unspoken yet powerful demand for governmental action, pushing for policies to manage and mitigate this health concern more effectively.
- **Influence of External Actors (e.g. donors)**: Financial incentives can exert a profound influence on policy priority-setting, driven by donor influence and government interests. Donors leverage funding to impact governmental agendas, while financial constraints shape governments' ability to champion specific topics (8, 46). This interplay reshapes policy trajectories, resource allocation and overall power dynamics. The influence of financial incentives on policy priority-setting reflects the interests of donors in influencing governmental agendas and the interests of governments in obtaining necessary resources as observed in Ghana's health priorities influenced by donor support (8). In this example, donors effectively used financial support as leverage to gain access to the policy agenda, influencing both the agenda's content and the prioritization of issues in Ghana (8). In a context of high donor dependency, new donor rules affect the composition and structure of actors in the networks, which enable the entry and dissemination of new ideas and shifts in the overall balance of interest power ultimately leading to policy change (24, 47). In Jordan, lack of funding led the UN High Commissioner for Refugees (UNHCR) to stop its support to the government delivery of healthcare services to Syrian refugees (47). This resulted in the government changing its refugee healthcare policy from allowing refugees to access Jordanian healthcare services on the same basis as nationals to giving refugees the same terms of access as uninsured Jordanians at a cost (47).
- **Policy Champions and Entrepreneurs:** Policy champions are individuals working in the policy arena who play a pivotal role in highlighting and elevating specific issues to policy prominence. They can be

high-level government officials such as prime minister and speaker of the parliament, representatives of international organizations, mid-level policy entrepreneurs and civil society activists (26, 31, 48-51). While policy champions focus on advocating for specific policies or causes, policy entrepreneurs strategically identify and promote new policy ideas, leveraging their networks and resources to influence the policy agenda. Both policy champions and entrepreneurs not only advocate for issues but also drive collective action and guide their implementation (7, 10, 13, 51, 52). For instance, in Honduras, representatives from the Pan American Health Organization (PAHO) acted as political entrepreneurs, publicizing critical health issues to convince key officials and collaborating with donors to mobilize the health system for the safe motherhood cause (51). In Lebanon, amidst the divisive context of cannabis, the parliament speaker spearheaded the initiative for medical use legalization, demonstrating the influence of policy champions in advancing specific topics (13, 52). Furthermore, the case of Canada highlights the influence of top-level leadership in policy transformation. The prioritization of neurodevelopmental disorders on the national agenda was significantly influenced by Prime Minister Justin Trudeau's call for a collaborative national autism strategy (10).

- **Policy Community Cohesion*:*** This factor involves the degree of cohesion among policy actors within a community, such as policymakers, media, international organizations, the public, and advocacy coalitions (10, 13, 53, 54). Cohesion among these stakeholders can either facilitate or hinder the prioritization of issues (10, 13, 53, 54). Advocacy coalitions is another form of cohesion; it can achieve a uniform voice and a common ground of values, policy aims, and implementation through negotiation (25, 26, 31, 35, 40, 49, 51, 55-59). In Mozambique, cohesive collaboration among various health stakeholders facilitated the effective design and implementation of policies for retaining rural health workers (54). While in Sri Lanka, a coalition between the Ministry of Women and women's NGOs was instrumental in advocating for and successfully passing the Prevention of Domestic Violence Act Bill (7).

- **Role of Knowledge Brokers:** Knowledge brokers contribute to framing the discussion around a topic, advocating for particular priority setting and resource allocation frameworks. This in turn can shape ideas and influence the discourse on national agendas (28, 40, 43, 57, 60-62). While these groups may have a less direct stake than other interest groups in the outcomes of particular priority setting decisions, their work will have political impact if they advocate – as policy entrepreneurs particular priority setting and resource allocation frameworks (1, 40, 57, 61). In particular, knowledge brokers can play a role in framing the discussion around a topic and can push and keep ideas on the national agenda. For example, in Lebanon, knowledge brokers intervened in the debate over the salt fluoridation law. They compiled an evidence-based brief to clarify and address the controversy, thereby playing a central role in informing the discourse (18, 43). Similarly, in Canada, the influence of knowledge brokers was evident in the formation of the British Columbia Code. Through their engagement in shaping discussions, knowledge brokers were instrumental in guiding the policy's development and eventual implementation (57).

3. Ideas

The "Ideas" category delves into the intricate landscape of policy prioritization, where various cognitive and intellectual forces intersect to shape the trajectory of issues on the policy agenda. Ideas, encompassing problem recognition and framing, guide decision-makers toward specific policy domains. Ideas encompass beliefs about what is (research knowledge) and views about what ought to be (values), affecting the prioritization of issues for knowledge translation. Factors under this category include political commitment, issue type, problem recognition, framing, availability of policy options, and equity implications.

- **Political Will and Commitment:** Political will and commitment emerged as critical facilitating factors in the prioritization and advancement of policy issues for knowledge translation — garnering support and resources for an issue (10, 13, 19-23, 42, 63-69). Political will reflects the leaders' intent and prioritization of issues for KT products, while political commitment is the manifestation of this will through concrete actions such as allocating governmental resources, shaping legislative and policy actions, and commissioning policy briefs that reflect policy priorities and societal needs (12, 13, 19, 20, 22, 23, 42, 53, 63-67, 69). For example, the Ministry of Health in Slovenia, driven by its commitment to address the issue of antibiotic resistance in long-term care facilities, directly commissioned a brief providing evidence-based solutions for consideration (70). Similarly, the UK government's commitment to supporting parental relationships was evidenced by a substantial increase in funding to £70 million, underscoring a strong dedication to this social issue (66). Moreover, Estonia's government initiative to assess and regulate the sales of energy drinks demonstrates their commitment to public health, particularly in protecting children's access to potentially harmful products (65).

- **Alignment with National Agendas and Goals:** The alignment of issues with a nation's strategic goals and plans significantly enhances their prioritization, creating fertile ground for effective knowledge translation to shape policy development and implementation. (10, 11, 22, 53, 54). Key mechanisms of influence for this alignment include the facilitation of knowledge translation and evidence utilization, as well as the creation of opportunities, or 'policy windows,' where timely and relevant issues can be addressed more effectively within the national agenda framework. In Mozambique, the alignment of rural area retention strategies with the third strategic objective of the National Plan for Health Human Resources Development exemplifies how national strategic objectives facilitate specific policy implementations (54). In Ontario, the government's announcement of a second health teams cohort was seized as a strategic opportunity to implement changes aimed at improving hospital to home transitions for older adults, showcasing the use of national agenda alignment to drive stakeholder-desired changes (11).
- **Issue Type**: The nature of the issue itself shapes political priority. Some issues are intrinsically easier to promote than others. The classification of policy issues as either salient or contestable can play a pivotal role in their prioritization. Salient issues, affecting a wide population or challenging the status quo, often demand immediate governmental response (11, 13, 19, 42, 43, 52, 65, 67, 71-78). Immediate concerns drive urgency, compelling swift attention and decisive action, triggering an instant need for evidence-based information(11). In contrast, contestable issues are marked by controversy - whether between actors, over how issues are framed, or over doubts about evidence or interventions-necessitating evidence to guide decisions (13, 52, 79, 80). Topics like cannabis legalization, taxation laws, and abortion exemplify contestable issues, while urgent matters like pandemic responses and disasters symbolize salient topics. For instance, the urgency of hospital to home transitions in Canada became a salient topic due to ongoing provincial initiatives and the exigencies of the COVID-19 pandemic, creating a critical context for policy action (11).
- **Problem Recognition:** A fundamental factor in shaping topic prioritization is the recognition and acknowledgment of an existing problem, often driven by the presence of evidence that underscores the issue's significance. This recognition is buoyed by credible indicators, research evidence, and data, which effectively bolster the case for addressing the problem and guide its prioritization (10, 44, 70, 72, 81, 82). Problem recognition mechanism of influence is rooted in creating a need to rectify the identified problem, create urgent demand and generate demand for action (83). When backed by credible and conspicuous facts, an urgent issue's discourse wields considerable influence, compelling a response. Problems easily measured are more likely to gain political support than ones that are not, as policymakers and advocates will have information to confirm the severity and monitor progress (10,

44, 70, 72, 81, 82). Illustrative examples can be seen in various global contexts. In nations such as Bhutan, East Timor, Rwanda, Laos, Maldives, Mongolia, and Cambodia, high maternal mortality ratios served as a critical indicator. This alarming data sparked political alarm, convincing decision-makers of the issue's severity and urgency, and consequently cementing its significance on the national policy agenda (84). Similarly, in Europe, the substantial economic burden of hospitalization costs prompted a reassessment of hospital ownership and governance structures. This case demonstrates how economic data and its implications can drive policy discussions and lead to significant reforms (85).

- **Framing:** The way an issue is framed can influence its perceived importance and urgency. This factor delves into how policy issues are presented and defined, employing social, psychological, and cultural concepts to imbue topics with meaning and interpretation. This dynamic process, facilitated by various stakeholders like evidence generators, enactors, intermediaries, and advocacy coalitions, serves to emphasize the problem's significance, offer diverse perspectives, and underscore reasons for its policy agenda inclusion (5, 8, 28, 86-90). Policy narratives, essentially persuasive stories, are also constructed by policy actors to advance their goals (91). The process of framing adjusts the problem's lens, links the issue with shared values to heighten its importance, and provides rationales for policymakers to address it (21, 52, 63, 67, 71, 92, 93). By emphasizing specific aspects or using particular language, advocates can shape the narrative around an issue, framing can shape public understanding and perception, influencing how individuals and policymakers interpret and prioritize the issue. Frames that resonate internally unify policy communities by providing a common understanding of the definition of, causes of and solutions to the problem while frames that resonate externally move critical audiences to action, particularly the political leaders who control the resources that initiatives need (45, 93, 94). For instance, the varied framing of HIV/AIDS as a public health problem, a development issue, a humanitarian crisis, and a security threat illustrates the multifaceted impact of framing on policy perception and action (69). In Ethiopia, framing the challenges in malaria elimination as impediments to achieving national and global goals created a sense of urgency and pressure for policymakers, emphasizing the need to address expertise shortages (63).
- **Availability of Alternative Policy Options:** The availability of feasible policy options plays a role in the prioritization of pressing issues (12, 13, 22, 23, 42, 63, 65, 66, 72). Problems with relatively simple, feasible, and evidence-based solutions will be easier to promote than those without these features, as policymakers prefer to devote resources to issues that they think they can address effectively and cheaply (22, 65, 94) Furthermore, linking the proposed solutions with existing policy principles that are widely supported or with the value or ideological thinking of the most influential groups can reduce resistance and increase receptiveness to prioritize address the issue (95). For example, in Turkey, the revision of legislative measures to reduce high trans fatty acid consumption in food products demonstrates the impact of having readily available policy options on addressing public health issues (22). Similarly, the strategy employed by the Netherlands to tackle health disparities highlights the effectiveness of aligning proposed solutions with widely accepted policy principles (95).
- **Shifts in Political Discourse:** Shifts in political discourse, characterized by changes in public opinion, societal values, and the language and narratives used in policy discussions, play a significant role in shaping policy agendas. Such shifts can bring new issues to the fore, alter public perceptions, and catalyze a demand for policy changes, thereby influencing the prioritization of certain topics. The evolution of political discourse reflects the dynamic nature of societal values and public opinion, which are pivotal in determining the trajectory of policy development. An illustrative case can be observed in the United Kingdom, where there have been growing discussions around the introduction of charging mechanisms for overseas patients receiving healthcare services (96). This discourse has led to significant policy considerations, including the proposal that overseas patients pay upfront for their healthcare services. Amidst the political rhetoric surrounding the alleged abuse of the healthcare system

by ‘freeloading’ health tourists, this policy debate exemplifies the power of populist appeals in shaping public policy. The discourse of the ‘other’ as a beneficiary of unmerited healthcare services has sparked discussions that challenge the principle of universal healthcare provision by the National Health Service (NHS) (96).

- **<u>Equity and Health Concerns</u>**: The anticipated outcomes of addressing a specific policy issue, particularly their potential effects on health, equity, and the economy, drives prioritization (18, 97). The foreseen benefits and consequences stimulate the demand for evidence to inform decision-making on specific priorities and propelling specific issues to the forefront (63, 82, 97). For example, in Canada, the COVID-19 pandemic's unequal impact on residents of long-term care homes prompted the government to initiate targeted responses (97). This action highlights the critical importance of expected impacts, such as equity in health outcomes and the broader social benefits, in driving policy decisions.

4. External events that affect political context

This category underscores the transformative role of external events in shaping policy priorities and highlights the delicate interplay between political landscapes and socioeconomic conditions. Factors under this category include changes in socioeconomic conditions, focusing events, geopolitical developments and global trends.

- **<u>Changes in Socioeconomic Conditions</u>**: Socioeconomic shifts wield considerable influence over policy agendas, dictating which issues rise to prominence and demand urgent attention (13, 52, 77). Economic turmoil can exacerbate existing challenges, amplifying issues like the absence of emergency preparedness plans (77) and straining healthcare sectors (78). Lebanon's economic crisis and budget deficits acted as catalysts for policy innovation, exemplified by the consideration of excise taxation as a means to address financial shortfalls (52).
- **<u>Focusing Events</u>**: Focusing events have emerged as pivotal agents in reshaping policy agendas by directing attention to previously overlooked issues (11, 73, 77, 78, 92, 97). These events, characterized by their extensive impact, tangible harm, novelty, and rarity, possess the potential to trigger policy change, driven by the narrative they construct. (13, 42, 63, 77, 92). The COVID-19 pandemic serves as a prime example of such an event, as it brought critical focus to healthcare sectors, particularly in areas like long-term care and hospital-to-home transitions(97). This global health crisis underscored the need for robust healthcare systems and effective patient care strategies, propelling these topics to the forefront of policy discussions. On the other hand, the influx of Syrian refugees into countries like Lebanon and Jordan had a profound impact on maternal health services within these host countries (73). This situation highlighted significant healthcare gaps and underscored the necessity for targeted policy interventions to address the health needs of both the refugee and local populations.
- **<u>Geopolitical Developments</u>**: Geopolitical events, such as global economic trends, and regional developments, changes in power dynamics between states, and conflicts influence the decisions and actions of policymakers, and thus, can impact the priority-setting process. For example, a global economic recession or a regional conflict may necessitate a shift in policy priorities since they have ripple effects that extend beyond borders, catalyzing the emergence of new priorities (98).
- **<u>Technological Advancements and Innovations:</u>** Technological innovations often highlight gaps in current healthcare systems and practices, prompting a reevaluation of priorities. For instance, the advent of telemedicine and digital health platforms has underscored the need for policies that address digital healthcare delivery, data security, and patient privacy (99). As these technologies become integral to healthcare provision, they necessitate a shift in policy focus towards ensuring equitable access, safeguarding patient data, and integrating new technologies into existing health systems (99). An

illustrative example of how technological innovation impacts policy prioritization is seen in the response to the COVID-19 pandemic (100). The rapid development of mRNA vaccine technology not only provided a solution to a global health crisis but also shifted national and international health policy priorities towards vaccine research and development, vaccine distribution, and public health communication strategies (100).

- **<u>Global Trends:</u>** There are moments in time when global conditions align favorably for an issue, presenting strong opportunities to reach national political leaders (94). For instance, governments may need to prioritize healthcare, public health infrastructure, and emergency response in the face of global health threats (44, 53, 63, 68, 72). A second critical element is the global governance structure for the health sector in charge of enforcing international treaties, laws and declarations related to health (94). Global health initiatives such as the SDGs or forums (e.g. global UN conferences) shape policy priorities by opening unique policy windows. For example, Ethiopia's focus on malaria elimination is driven by global targets alignment with global agendas created political pressure to conform with international trends such as the SDGs and initiatives to fight malaria (63).

References

1. Smith N, Mitton C, Davidson A, Williams I. A politics of priority setting: Ideas, interests and institutions in healthcare resource allocation. Public Policy and Administration. 2014;29(4):331-47.
2. Astley J, Wake-Dyster W. Evidence-based priority setting. Australian Health Review. 2001;24(2):32-9.
3. Barasa EW, Molyneux S, English M, Cleary S. Setting healthcare priorities in hospitals: a review of empirical studies. Health policy and planning. 2015;30(3):386-96.
4. Valdebenito C, Kapiriri L, Martin DK. Hospital priority setting in a mixed public/private health system: a case study of a Chilean hospital. Acta bioethica. 2009;15(2):193-201.
5. Baker P, Friel S, Kay A, Baum F, Strazdins L, Mackean T. What enables and constrains the inclusion of the social determinants of health inequities in government policy agendas? A narrative review. International Journal of Health Policy and Management. 2018;7(2):101.
6. Oliver TR. The politics of public health policy. Annu Rev Public Health. 2006;27:195-233.
7. Colombini M, Mayhew SH, Lund R, Singh N, Swahnberg K, Infanti J, et al. Factors shaping political priorities for violence against women-mitigation policies in Sri Lanka. BMC international health and human rights. 2018;18(1):1-12.
8. Koduah A, van Dijk H, Agyepong IA. The role of policy actors and contextual factors in policy agenda setting and formulation: maternal fee exemption policies in Ghana over four and a half decades. Health Research Policy and Systems. 2015;13(1):1-20.
9. Hall PA, Taylor RC. Political science and the three new institutionalisms. Political studies. 1996;44(5):936-57.
10. Moat KA VP, Bhuiya AR, Ahmad A, Lavis JN. Creating a Pan-Canadian Learning Health

System for Neurodevelopmental Disorders. 2020.
11. Gauvin FP WK, Ganann R, Heald-Taylor G, Markle-Reid M, McAiney C, Lavis JN. Improving Hospital-To-Home Transitions for Older Adults with Complex Health and Social Needs in Ontario. 2020.
12. Beović B, Čižman M, Papst L, Pečavar B, Šubelj M, Dobrin PT. Antibiotic prescribing in long-term care facilities

for the elderly. 2018.
13. Hilal N B-KL, Ataya N, El-Jardali F. Legalizing Cannabis Cultivation: What we need to know & is Lebanon Ready? 2018.
14. Liverani M, Hawkins B, Parkhurst JO. Political and institutional influences on the use of evidence in public health policy. A systematic review. PloS one. 2013;8(10):e77404.
15. Gauvin F-P. Understanding policy developments and choices through the “3-i” framework: interests, ideas and institutions. 2014.
16. Raab J, Kenis P. Taking stock of policy networks: do they matter? Handbook of public policy analysis: Routledge; 2017. p. 213-26.
17. Fazekas M, Burns T. Exploring the complex interaction between governance and knowledge in education. 2012.
18. Akik C E-MC, Ghattas H, Obeid O, El-Jardali F. Informing Salt Iodization Policies in Lebanon to Ensure Optimal Iodine Nutrition. 2016.
19. MG. W. Examining the Effectiveness and Cost-effectiveness of Rehabilitation-care Models for Frail Seniors. 2013.
20. Waddell K GF, Mattison CA. Fostering K-12 Students’ Global Competencies. 2018.

21. Mattison CA MK, Scallan EM, Gauvin FP, Lavis JN. Reducing Emergency-department Usage in People with Inflammatory Bowel Disease in Provincial Health Systems in Canada. 2018.
22. Ari HO, Işlek, E, Özatkan, Y, Bilir, M K, Demirkesen Mert, I, Özcan Çetin, E H, Yildirim, H H. Reducing the consumption of trans-fats and their negative impacts on health in Turkey. 2021.
23. Tirdea M CA, Obreja G. Informing amendments to the alcohol control legislation directed at reducing harmful use of alcohol in the Republic of Moldova. 2019.
24. Shearer JC, Abelson J, Kouyaté B, Lavis JN, Walt G. Why do policies change? Institutions, interests, ideas and networks in three cases of policy reform. Health policy and planning. 2016;31(9):1200-11.
25. Rajataramya B, Fried B, van der Putten M, Pongpanich S. Autonomous public organization policy: a case study for the health sector in Thailand. Southeast Asian J Trop Med Public Health. 2009;40(5):1092-102.
26. Baker P, Gill T, Friel S, Carey G, Kay A. Generating political priority for regulatory interventions targeting obesity prevention: an Australian case study. Social Science & Medicine. 2017;177:141-9.
27. Shiffman J. Generating political priority for maternal mortality reduction in 5 developing countries. American journal of public health. 2007;97(5):796-803.
28. Votruba N, Grant J, Thornicroft G. The EVITA framework for evidence-based mental health policy agenda setting in low-and middle-income countries. Health policy and planning. 2020;35(4):424-39.
29. Lavis JN. Research, public policymaking, and knowledge-translation processes: Canadian efforts to build bridges. Journal of Continuing Education in the Health Professions. 2006;26(1):37-45.
30. Berman J, Mitambo C, Matanje-Mwagomba B, Khan S, Kachimanga C, Wroe E, et al. Building a knowledge translation platform in Malawi to support evidence-informed health policy. Health research policy and systems. 2015;13(1):1-5.
31. Colombini M, Mayhew SH, Lund R, Singh N, Swahnberg K, Infanti J, et al. Factors shaping political priorities for violence against women-mitigation policies in Sri Lanka. BMC International Health and Human Rights. 2018;18(1).
32. Walt G, Gilson L. Can frameworks inform knowledge about health policy processes? Reviewing health policy papers on agenda setting and testing them against a specific priority-setting framework. Health Policy Plan. 2014;29 Suppl 3:iii6-22.
33. Carriedo A, Lock K, Hawkins B. Policy Process And Non-State Actors' Influence On The 2014 Mexican Soda Tax. Health Policy Plan. 2020;35(8):941-52.
34. Hinchcliff R, Poulos R, Ivers RQ, Senserrick T. Understanding novice driver policy agenda setting. Public Health (Elsevier). 2011;125(4):217-21.
35. Harris P, Kent J, Sainsbury P, Marie-Thow A, Baum F, Friel S, et al. Creating 'healthy built environment' legislation in Australia; a policy analysis. Health Promot Internation. 2018;33(6):1090-100.
36. Varghese J, Blankenhorn A, Saligram P, Porter J, Sheikh K. Setting the agenda for nurse leadership in India: What is missing Lucy Gilson. International Journal for Equity in Health. 2018;17(1).
37. Thow AM, Apprey C, Winters J, Stellmach D, Alders R, Aduku LNE, et al. Understanding the Impact of Historical Policy Legacies on Nutrition Policy Space: Economic Policy Agendas and Current Food Policy Paradigms in Ghana. Int. 2020;09:09.
38. Yapeng Z, Cheng JYS. The Emergence of Cyber Society and the Transformation of the Public Policy Agenda-Building Process in China*. China Review. 2011;11(2):153-81.
39. Onono MA, Rutherford GW, Bukusi EA, White JS, Goosby E, Brindis CD. Political prioritization and the competing definitions of adolescent pregnancy in Kenya: An application of the Public Arenas Model. PLoS ONE. 2020;15(9):e0238136.
40. Votruba N, Grant J, Thornicroft G. The EVITA framework for evidence-based mental health policy agenda setting in low- and middle-income countries. Health Policy & Planning. 2020;35(4):424-39.

41. Colombini M, Mayhew SH, Hawkins B, Bista M, Joshi SK, Schei B, et al. Agenda setting and framing of gender-based violence in Nepal: how it became a health issue. Health policy and planning. 2016;31(4):493-503.
42. Gauvin FP WK, Lavis JN. Fostering an Organizational Culture Supportive of Evidence-informed Policymaking. 2017.
43. Fadlallah R E-JF, Ghaddar F, Hamad L. Informing the Salt Fluoridation Law in Lebanon. 2015.
44. WHO. Eliminating trans fats in Europe. 2015.
45. Alikhani M, Vatankhah S, Gorji HA, Ravaghi H. How cancer supportive and palliative care is developed: Comparing the policy-making process in three countries from three continents. Indian Journal of Palliative Care. 2020;26(1):72.
46. Fischer SE, Strandberg-Larsen M. Power and agenda-setting in Tanzanian health policy: an analysis of stakeholder perspectives. International Journal of Health Policy and Management. 2016;5(6):355.
47. Bellamy C, Haysom S, Wake C, Barbelet V. The lives and livelihoods of Syrian refugees: a study of refugee perspectives and their institutional environment in Turkey and Jordan. HPG Commissioned Report London: ODI. 2017.
48. Colombini M, Mayhew SH, Hawkins B, Bista M, Joshi SK, Schei B, et al. Agenda setting and framing of gender-based violence in Nepal: how it became a health issue. Health Policy Plan. 2016;31(4):493-503.
49. Pelletier DL, Frongillo EA, Gervais S, Hoey L, Menon P, Ngo T, et al. Nutrition agenda setting, policy formulation and implementation: lessons from the Mainstreaming Nutrition Initiative. Health Policy & Planning. 2012;27(1):19-31.
50. Ha BT, Mirzoev T, Mukhopadhyay M. Shaping the Health Policy Agenda: The Case of Safe Motherhood Policy in Vietnam. Int. 2015;4(11):741-6.
51. Shiffman J, Stanton C, Salazar AP. The emergence of political priority for safe motherhood in Honduras. Health Policy and Planning. 2004;19(6):380-90.
52. Hilal N FR, Jamal D, El-Jardali F. How can excise taxation be utilized to improve health outcomes in Lebanon? 2017.
53. Mwape L MP. Strengthening the health system

for mental health in Zambia. 2011.
54. Mbofana FS SC, Machatine G. The retention of health workers in rural and remote areas in Mozambique. 2012.
55. Heller O, Somerville C, Suggs LS, Lachat S, Piper J, Aya Pastrana N, et al. The process of prioritization of non-communicable diseases in the global health policy arena. Health Policy Plan. 2019;34(5):370-83.
56. Okeke C, Manzano A, Obi U, Etiaba E, Onwujekwe O, Mirzoev T, et al. Exploring mechanisms that explain how coalition groups are formed and how they work to sustain political priority for maternal and child health in Nigeria using the advocacy coalition framework. Health Res Policy Syst. 2021;19(1):26.
57. Kamieniecki S. Testing alternative theories of agenda setting: Forest policy change in British Columbia, Canada. Policy Studies Journal. 2000;28(1):176-89.
58. Mukanu MM, Zulu JM, Mweemba C, Mutale W. Responding to non-communicable diseases in Zambia: A policy analysis. Health Research Policy and Systems. 2017;15(1).
59. McCullum C, Pelletier D, Barr D, Wilkins J. Agenda setting within a community-based food security planning process: the influence of power. J Nutr Educ Behav. 2003;35(4):189-99.
60. Agyepong IA, Adjei S. Public social policy development and implementation: a case study of the Ghana National Health Insurance scheme. Health policy and planning. 2008;23(2):150-60.

61. Agyepong IA, Adjei S. Public social policy development and implementation: a case study of the Ghana National Health Insurance scheme. Health Policy Plan. 2008;23(2):150-60.
62. Buse K, Mays N, Walt G. Making health policy: McGraw-hill education (UK); 2012.
63. Woyessa A KAaHM. Human Resource Capacity to Effectively Implement Malaria Elimination in Ethiopia. 2010.
64. Dibaba A HM, Ababor S, Assefa Y. Improving health care financing in Ethiopia. 2014.
65. Köhler K EM, Peil E, Sammel A, Uuetoa M, Villa. Reducing the consumption of sugar-sweetened beverages and their negative health impact in Estonia. 2016.
66. Stock LA, D; Molloy, D; Piergallini, I. INTER-PARENTAL RELATIONSHIPS,

CONFLICT AND THE IMPACTS OF

POVERTY. 2017.
67. Parsons KH, C. Connecting food systems for co-benefits: How can food systems combine diet-related health with environmental and economic policy goals? 2018.
68. Saleh R, Nakkash, R., & El-Jardali, F. Promoting Effective School Policies for Childhood Overweight & Obesity Prevention in Lebanon. 2019.
69. Wilson MG MC, Gao C, Scallan EM, Kendall CE, Lavis JN. Enhancing the Delivery of Comprehensive Care for People Living with HIV in Canada. 2019.
70. Kostova NB, G; Stavrikj, K. Promoting appropriate use of antibiotics in hospitals to contain antibiotic resistance in North Macedonia. 2020.
71. Nabudere H, Asiimwe D, Amandua J. Improving access to skilled attendance at delivery: a policy brief for Uganda. International journal of technology assessment in health care. 2013;29(2):207-11.
72. Erica Richardson JZ, Ellen Nolte. National Diabetes Plans in Europe: What lessons are there for the prevention and control of chronic diseases in Europe? 2016.
73. El-Jardali F, Abou Samra, C., Hemadi, N., Bawab, L., El Kak, F. Reducing Preventable Preterm Deliveries among Syrian Refugees in Lebanon. 2017.
74. Waddell S CA. Social & emotional learning: supporting children and young people’s mental health. 2017.
75. Mattison CA MK, Waddell K, Lavis JN. Optimizing Patient and Family Transitions from Cancer Treatment to Primary- and Community-care Supports in Canada. 2018.
76. Veazie S BD, Peterson K, Anderson J. Video Telehealth for Primary Care and Mental Health Services. 2019.
77. Bou-Karroum L FR, Jabbour M, Daher N, El-Jardali F. Rapid Guide to Support Health and Social Care Systems in Response to Beirut Explosion. 2020.
78. Hilal N SR, Hamadeh R, Abou Samra C, El-Jardali F. Integrating COVID-19 Vaccination into the Primary Health Care Network in Lebanon. 2021.
79. Moat KA, Lavis JN, Abelson J. How contexts and issues influence the use of policy-relevant research syntheses: a critical interpretive synthesis. The Milbank Quarterly. 2013;91(3):604-48.
80. Walt G, Gilson L. Can frameworks inform knowledge about health policy processes? Reviewing health policy papers on agenda setting and testing them against a specific priority-setting framework. Health policy and planning. 2014;29(suppl_3):iii6-iii22.
81. Vogler SP, V; Panteli, D. Ensuring access to medicines: How to redesign pricing, reimbursement and procurement? 2018.
82. Guindon GE FT, Buckley G, Montreuil A, Lavis JN, Wilson MG. E. Addressing Area-level Disparities in Prices of Tobacco and Vaping Products in Ontario and Québec. 2019.
83. Babaey F, Raessi P, Ravaghi H. Agenda setting analysis for maternal mortality reduction: Exploring influential factors using Kingdonâ s stream model Analyse de la dÃ© finition des enjeux

prioritaires pour la rÃ© duction de la mortalitÃ© maternelle: Exploration des facteurs influents Ã l'aide du modÃ¨ le des flux de Kingdon. Eastern Mediterranean Health Journal. 2019;25(3):160-71.
84. Hogan D. National, regional, and global levels and trends in maternal mortality between 1990 and 2015 with scenario-based projections to 2030: a systematic analysis by the United Nations Maternal Mortality Estimation Inter-Agency Group. vol. 387, no. 10017. 2017. Europe PMC free article][Abstract][Google Scholar]. 2017:462-74.
85. Rechel BD, A; Saltman, R. What is the experience of decentralized hospital governance in Europe? 2018.
86. Harris P, Kent J, Sainsbury P, Marie-Thow A, Baum F, Friel S, et al. Creating 'healthy built environment' legislation in Australia; a policy analysis. Health promotion international. 2018;33(6):1090-100.
87. Koduah A, Gyansa-Lutterodt M, Hedidor GK, Sekyi-Brown R, Asiedu-Danso M, Asare BA, et al. Antimicrobial resistance national level dialogue and action in Ghana: setting and sustaining the agenda and outcomes. One health outlook. 2021;3(1):1-12.
88. Liu Y, Chan RHy. The Framework of Crisis-Induced Agenda Setting in China. Asia & the Pacific Policy Studies. 2018;5(1):18-33.
89. Shiffman J, Beer T, Wu Y. The emergence of global disease control priorities. Health Policy and Planning. 2002;17(3):225-34.
90. Townsend B, Strazdins L, Harris P, Baum F, Friel S. Bringing in critical frameworks to investigate agenda-setting for the social determinants of health: Lessons from a multiple framework analysis. Social Science & Medicine. 2020;250:112886.
91. McBeth MK, Lybecker DL. The narrative policy framework, agendas, and sanctuary cities: The construction of a public problem. Policy Studies Journal. 2018;46(4):868-93.
92. El-Jardali F, El Bawab L., Fadlallah, R. Addressing Medical Errors in the Lebanese Healthcare System. 2016.
93. wellbeing Ww. Briefing: What do we know about tackling loneliness? 2018.
94. Shiffman J. A social explanation for the rise and fall of global health issues. Bulletin of the World Health Organization. 2009;87(8):608-13.
95. Schmidt M, Joosen I, Kunst AE, Klazinga NS, Stronks K. Generating political priority to tackle health disparities: a case study in the Dutch city of The Hague. American Journal of Public Health. 2010;100(S1):S210-S5.
96. Speed E, Mannion R. The rise of post-truth populism in pluralist liberal democracies: challenges for health policy. International journal of health policy and management. 2017;6(5):249.
97. Gauvin FP WM, DeMaio P, Alam S, Drakos A, Lavis JN. Identifying and Harnessing the Potential of Technology in Long-term Care Settings

in Canada. 2021.
98. Alvarez E, Lavis JN, Brouwers M, Carmona Clavijo G, Sewankambo N, Solari L, et al. Developing evidence briefs for policy: a qualitative case study comparing the process of using a guidance-contextualization workbook in Peru and Uganda. Health research policy and systems. 2019;17(1):1-11.
99. European Commission. The Use of Big Data in Public Health Policy and Research. Background information document. Retrieved from https://health.ec.europa.eu/system/files/2016-11/ev_20141118_co07b_en_0.pdf. 2014.
100. Daems R, Maes E. The race for COVID-19 vaccines: Accelerating innovation, fair allocation and distribution. Vaccines. 2022;10(9):1450.
